# A Bayesian approach for exploring the effects of household income on self-reported mental health measures during the COVID-19 pandemic

**DOI:** 10.1371/journal.pmen.0000691

**Published:** 2026-08-25

**Authors:** Simon Busch-Moreno, Xiao Fu, Etienne B. Roesch

**Affiliations:** 1 Center for the Well-Being and Development of Adolescents and Children in the Digital Age (BAND), Pontifical Catholic University of Chile, Santiago, Chile; 2 Institute of Science and Innovation in Medicine (ICIM), University of Development, Santiago, Chile; 3 Department of Computer Science and Engineering, College of Engineering, Southern University of Science and Technology, Shenzhen, China; 4 School of Psychology and Clinical Language Sciences, University of Reading, Reading, United Kingdom; 5 Centre for Integrative Neuroscience and Neurodynamics (CINN), University of Reading, Reading, United Kingdom; Département des Yvelines / INSERM, FRANCE

## Abstract

The relationship between income and mental health is well-established yet complex, as both can be confounded by age, gender, and other demographic factors. To clarify these dynamics, this study examines the effects of age and income on symptoms of depression and generalised anxiety across female and male genders during the COVID-19 pandemic in the UK. Data from Wave-1 and Wave-6 of the COVID-19 Psychological Research Consortium (C19PRC) study were analysed using Bayesian hierarchical ordered-logistic mediation models. Results showed that older age was linked to less depression and anxiety in both genders. Higher income was linked to better mental health in males in both waves. For females, however, higher income was only weakly linked to more symptoms early in the pandemic, but by Wave‑6, it had become a substantially protective factor for their mental health. Additionally, males reported better mental health than females across most income groups, except at the lowest income group, where both genders experienced comparable distress. Using robust inferential statistical models, our findings explain the nuanced relationships between socioeconomic factors and mental health across genders during a public health crisis. This evidence can inform targeted mental health policies and economic support programs for future emergencies.

## 1. Introduction

The literature consistently shows a strong relationship between socioeconomic status and mental health, including a negative association between income and mental health [1], and between wealth and mental health [2]. Consistent with this pattern, recent studies in the United States have shown a beneficial effect of income on mental health [3] and a negative association between wealth (financial assets) and self-reported measures of depression and anxiety [4]. Similarly, recent literature from China indicates that high socioeconomic welfare is associated with a lower risk of depression [5] and that provincial-level income inequality is positively associated with depressive symptoms [6]. More broadly, the association between income inequality and depression has been noted early on (e.g., [7]; [8] and has since been consistently observed in the literature [9]. In Europe, this association has been observed both at the individual and country levels, where psychometric measures of depression negatively correlated with income quintiles and positively correlated with country-level income inequality [10]. Illustrating the role of policy-mediated income changes, a study in the United Kingdom (UK) has observed that the reduction of housing support increased the prevalence of depression on low-income people [11]. Finally, more recent evidence suggests that the mental health impacts of COVID-19 were disproportionately associated with low annual income, amongst other factors [12].

Even though rich datasets have been recorded during the COVID-19 pandemic, with both income and mental health [13], to the best of our knowledge these have not been used to address the association between mental health and income directly via a causal model, where income may be a mediator of other factors, such as age. In the present study we explore the relationship between gender, age, income, and mental health using data from the COVID-19 Psychological Research Consortium Study (C19PRC, see: [13]. The C19PRC dataset provides measures of age (years old), weekly household income (5 levels), and several mental health measures, including the Patient Health Questionnaire-9 (PHQ-9) and the Generalised Anxiety Disorder-7 (GAD-7) questionnaire. Both are self-report psychometric tests: PHQ-9 oriented to measure depressive (major depression or major depressive disorder: MDD) symptoms [14], and GAD-7 to measure generalised anxiety disorder (GAD) symptoms [15]. Both have been used in previous literature (e.g., [4] to explore associations between mental health and wealth. In the present case, we will explore the influence of age and income, across gender, on both measures.

Previous literature has observed an association between income and wealth, often described as a complex relationship, where wealth inequality may differ across locations (e.g., regions, countries, places), depending upon home ownership, real-estate price, or household savings [16]. Hence, higher income in countries with low home ownership and high real estate prices may be related with higher savings as a strategy for household wealth accumulation. For instance, in the UK the top 20% of richest households holds both financial and real-estate wealth [16]. Although in OECD countries (like the UK) wealth inequality is higher than income inequality [16], having a history of higher income may be associated with more financial stability at the individual level. Hence, we still expect to observe effects of household income on mental health. Income effects on mental health have been widely demonstrated in the previous literature (see [1], but they have not been systematically explored, using bespoke causal models, during periods of global high stress. Such periods, like the COVID-19 pandemic, are characterised by unusually high levels of uncertainty and emotional strain. In this study, our goal is to propose a methodological framework for understanding the effects of income on mental health as a potential mediator of age and to distinguish that effect across two genders (female and male, due to lack of sufficient data on other genders). We apply a Bayesian hierarchical ordered-logistic mediation model to understand the effects of age mediated by income across gender on both PHQ-9 and GAD-7 item by item.

Because our predictor is household income, the direct influence of age and gender of individuals is likely to be limited, as gender-related (e.g., pay gaps) and age-related (e.g., generational accumulation) disparities may not be directly reflected at household income levels. However, we expect overall disparities on mental health outcomes. For instance, during the COVID-19 pandemic, women took on a disproportionate share of caregiving and household tasks [17]. This is particularly relevant when considering the higher prevalence of mental health problems among women (e.g., [18–20]).

Regarding age, recent studies indicate that general wellbeing measures tend to increase with income but to decrease at older ages [21]. However, previous literature indicates that measures of depression, anxiety, or distress tend to decrease with age [22]. One possible explanation is that household income may mediate this relationship. Salary is a key component of household income for working-age adults, and it tends to increase with age, which in turn enhances saving capacity and reduces financial distress. Thus, age, household income and mental health may have a more complex relationship. Age is also an important biological and cultural factor influencing mental health. Its effects, however, are context-dependent: age may protect against or increase the risk of mental health problems, and this association is not necessarily linear (e.g., [21]. Although effects of age on income are not our primary focus, we consider them sufficiently important as to be addressed together with gender as secondary exposures.

In short, we expect a moderate negative effect of age on depression/anxiety measures mediated by income, where income should have a strong negative effect on depression/anxiety measures. That is, as age and income increase depression/anxiety measures should decrease (i.e., mental health should increase). We also expect overall gender disparities (with comparatively higher depression/anxiety for the female group). We emphasise, however, that the focus of the present study is on exploring the causal relationship (mediation or direct effect) of income on mental health.

## 2. Results

### 2.1 Depression results

**Fig 1:**
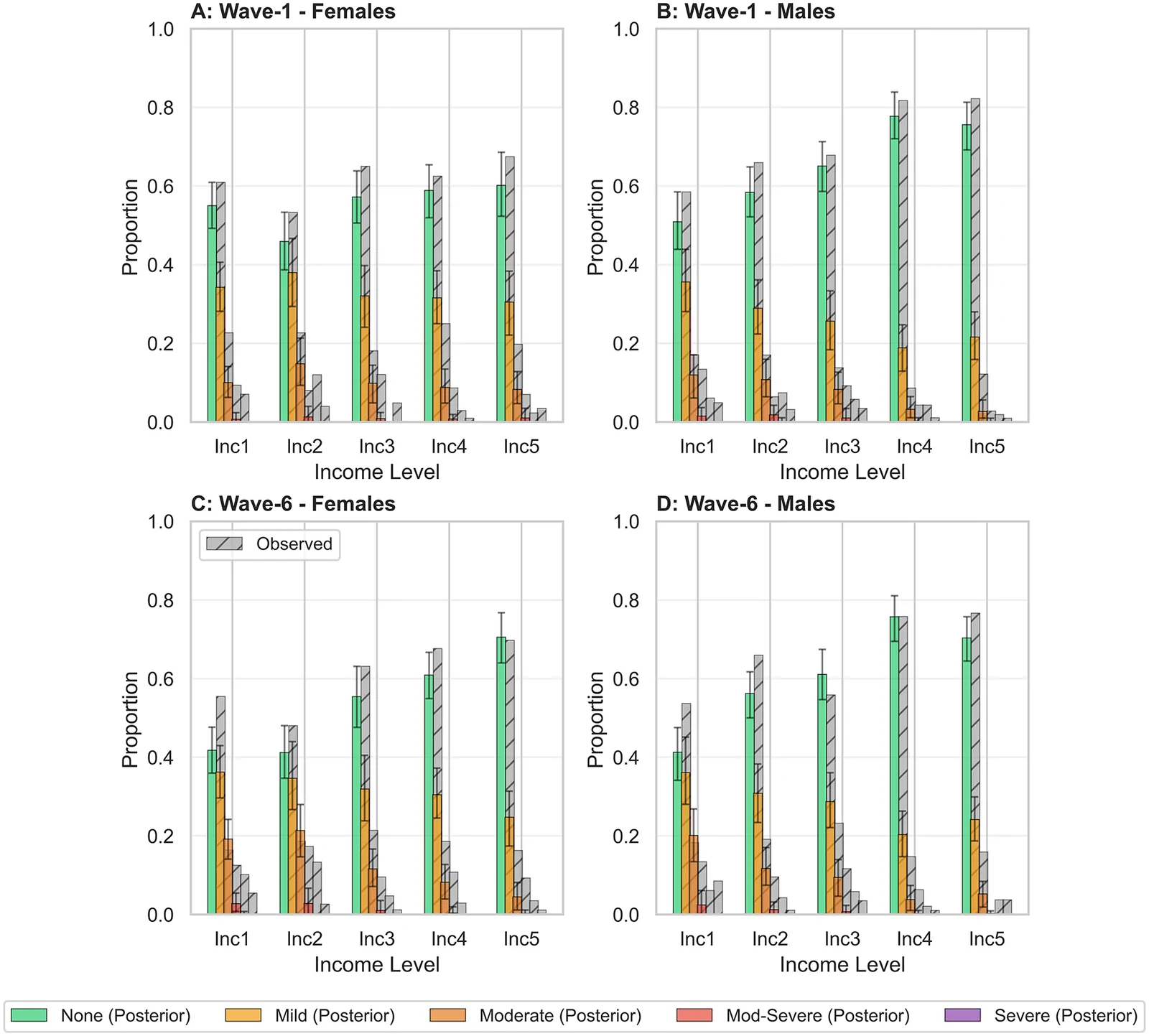
Posterior predictive distributions of PHQ-9 scores by income and depression severity. Bars represent posterior predictive means; error bar represent 90% HDIs (highest density intervals). Hatched bars show the observed proportion of responses within each PHQ-9 threshold.

Results from the hierarchical Bayesian mediation model for Wave-1 showed that, except for the lowest income group (Inc1), males in every other income group had a higher estimated probability (averaged across age) of scoring in the “none/minimal” category for depression symptoms compared to females (males: Inc1 = 51% [44%, 59%], Inc2 = 58% [52%, 65%], Inc3 = 65% [59%, 71%], Inc4 = 78% [72%, 84%], Inc5 = 76% [69%, 81%]; females: Inc1 = 55% [49%, 61%], Inc2 = 46% [39%, 53%], Inc3 = 57% [51%, 64%], Inc4 = 59% [52%, 65%], Inc5: 60% [52%, 69%]) (Fig 1).

**Fig 2:**
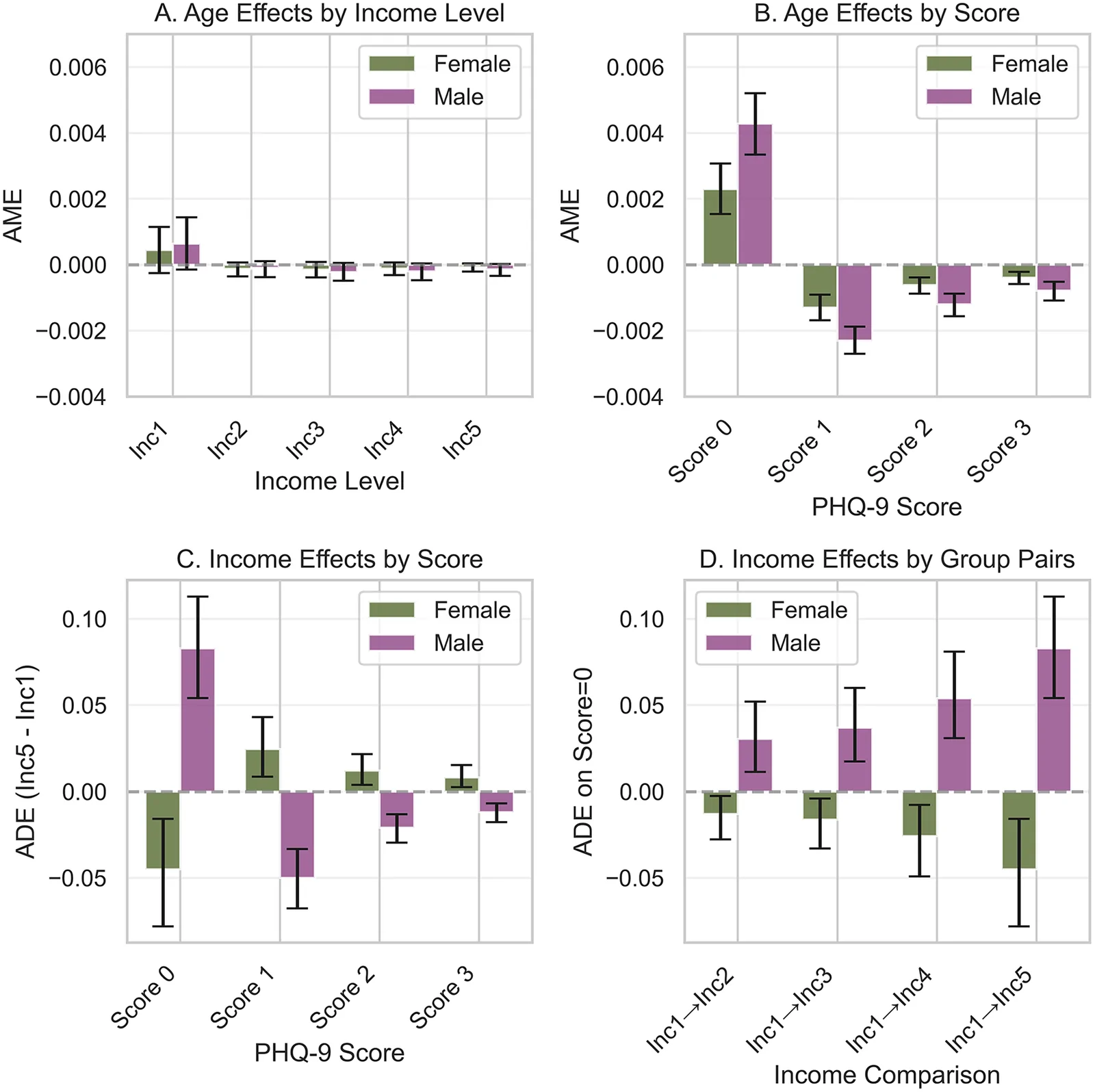
Summary of effects on PHQ-9 scores during Wave-1. (A) Effects of age on weekly household income levels; effects are presented as average marginal effects (AMEs) for males and females across the five income levels; each AME value represents unit percentage point change in income by year of age (18 years old minimum and 83 years old maximum). (B) Age AMEs on PHQ-9 four possible scores [“Not at all (0)”, “Several days (1)”, “More than half the days (2)”, “Nearly every day (3)”]; as before, AME represents the unit change in percentage by 1-year age increase. (C) Effects of income on PHQ-9 scores are expressed as average discrete effects (ADEs), which represent the ordered category probability difference between the lowest income group (Inc1 (£0-£300)) to the highest (Inc5 (£1,112+)) (i.e., Inc5 probability - Inc1 probability). (D) Effects of income on scoring 0 (“Not at all”) on any PHQ-9 item are expressed as ADEs, x-axis shows every increase from Inc1 (lowest income group) to every other income level.

Table 1 summarises averaged age and income effects from the causal model. Weekly household income and age showed strong associations with PHQ-9 outcomes in general, while the mediation of age by income appeared negligible. Among males, the probability of scoring 0 (answering “not at all”) on any single PHQ-9 item averaged across all 9 items was 8.2 percentage points higher in the highest income group (£1,112+) than in the lowest (£0–£300), averaged across the observed age range (18–83 years). In contrast, for females, this probability was 4.4 percentage points lower in the highest income group than in the lowest (Table 1 and Fig 2C and 2D). Age-related effects on scoring 0 on PHQ-9 items differed only modestly by gender: as age increased, the probability rose by 15.6 percentage points for females and 22.9 percentage points for males. As Table 1 summarises full averages, it displays small effect sizes relative to uncertainty. The causal model shows no evidence of income being a major mediator between age and depression regarding the observed sample.

**Table 1:** Wave-1 summary of effects on PHQ-9 (averaged over questions).

Effect	Gender	Mean	SD	5% HDI	95% HDI	Relationship
Mediator	Female	-0.044	0.058	-0.140	0.040	b:Income→Score
Mediator	Male	0.082	0.060	-0.014	0.182	b:Income→Score
Direct	Female	0.156	0.137	-0.049	0.406	c:Age→Score
Direct	Male	0.229	0.134	0.024	0.452	c:Age→Score
Indirect	Female	0.000	0.000	0.000	0.001	ab:Age→Income→Score
Indirect	Male	-0.001	0.001	-0.002	0.001	ab:Age→Income→Score
Total	Female	0.156	0.137	-0.053	0.403	c+ab
Total	Male	0.229	0.134	0.024	0.450	c+ab

Note: In this table, the income effect is defined as the age- and item-averaged probability of answering “Not at all” (score 0) on any PHQ-9 question for the highest income group (£1,112+) minus the corresponding probability for the lowest income group (£0–£300). The effect of age corresponds to the increase of the income- and item-averaged probability from the lowest age (18 years old) to the highest age (83 years old). HDI corresponds to the 90% highest density interval.

Fig 2 presents disaggregated results, which show the increases more clearly because the uncertainty is distributed across subgroups rather than concentrated in a single pooled estimate. In Wave-1, with increasing age, income level changed negligibly for both genders (Fig 2A). However, the probability of scoring 0 on PHQ-9 items increased, while the probability of scoring 1–3 decreased. Age effects for males were almost twice as high as for females (Fig 2B), although both were very small. The income effect on the probability of scoring 0 on PHQ-9 items, which was defined as the probability of the highest income group minus that of the lowest income group, was positive for males whereas negative for females (Fig 2C and Table 1), and this pattern held across all income groups when compared to the lowest income group (Fig 2D).

In Wave‑6, males in Income group 2, 3, and 4 showed higher probabilities than females of falling into the lowest PHQ‑9 category (minimal or no depression), but this probability for both genders were very close in the extreme income groups (i.e., Inc 1 and Inc 5) (males: Inc1 = 41% [34%, 48%], Inc2 = 56% [50%, 62%], Inc3 = 61% [55%, 67%], Inc4 = 76% [69%, 81%], Inc5 = 70% [64%, 76%]; females: Inc1 = 42% [36%, 48%], Inc2 = 41% [35%, 48%], Inc3 = 55% [48%, 63%], Inc4 = 61% [55%, 67%], Inc5 = 71% [64%, 77%]) (Fig 1). With respect to Wave-1, the probability of being categories in “minimal or none” depression level decreased for all income groups in males. However, among females, this probability decreased in the lower income groups (Inc1, 2 and 3) but increased in the higher income groups (Inc4 and 5) (Fig 1).

Table 2 summarises full averages across age or income groups. The results indicate that, for males, the probability of scoring 0 on PHQ-9 items for the highest income group was 6.5 percentage points higher than for the lowest income group. For females, this difference was 9.8 percentage points—in the same direction, though the pattern was reversed in the previous wave (Also see in Fig 2C and 2D). The age effect on the probability of scoring 0 on PHQ-9 items was larger for males (16.8 percentage points) than for females (9.8 percentage points), but the magnitude of this gender difference was similar to that observed in Wave-1. The indirect effect of age was also negligible in this wave (Table 2). Income does not show a major mediation effect in this sample either. Expanded summaries (by question) can be found in the supporting information of this study (Table A in S1 Appendix for Wave-1, Table B in S1 Appendix for Wave-6).

**Table 2:** Wave-6 summary of effects on PHQ-9 (averaged over questions).

Effect	Gender	Mean	SD	5% HDI	95% HDI	Relationship
Mediator	Female	0.098	0.062	0.008	0.198	b:Income→Score
Mediator	Male	0.065	0.057	-0.026	0.158	b:Income→Score
Direct	Female	0.098	0.120	-0.109	0.305	c:Age→Score
Direct	Male	0.168	0.115	-0.009	0.364	c:Age→Score
Indirect	Female	-0.001	0.001	-0.002	0.000	ab:Age→Income→Score
Indirect	Male	-0.001	0.001	-0.004	0.001	ab:Age→Income→Score
Total	Female	0.097	0.120	-0.110	0.304	c+ab
Total	Male	0.167	0.115	-0.010	0.363	c+ab

Note: In this table, the income effect is defined as the age- and item-averaged probability of answering “Not at all” (score 0) on any PHQ-9 question for the highest income group (£1,112+) minus the corresponding probability for the lowest income group (£0–£300). The effect of age corresponds to the increase of the income- and item-averaged probability from the lowest age (20 years old) to the highest age (84 years old). HDI corresponds to the 90% highest density interval.

**Fig 3:**
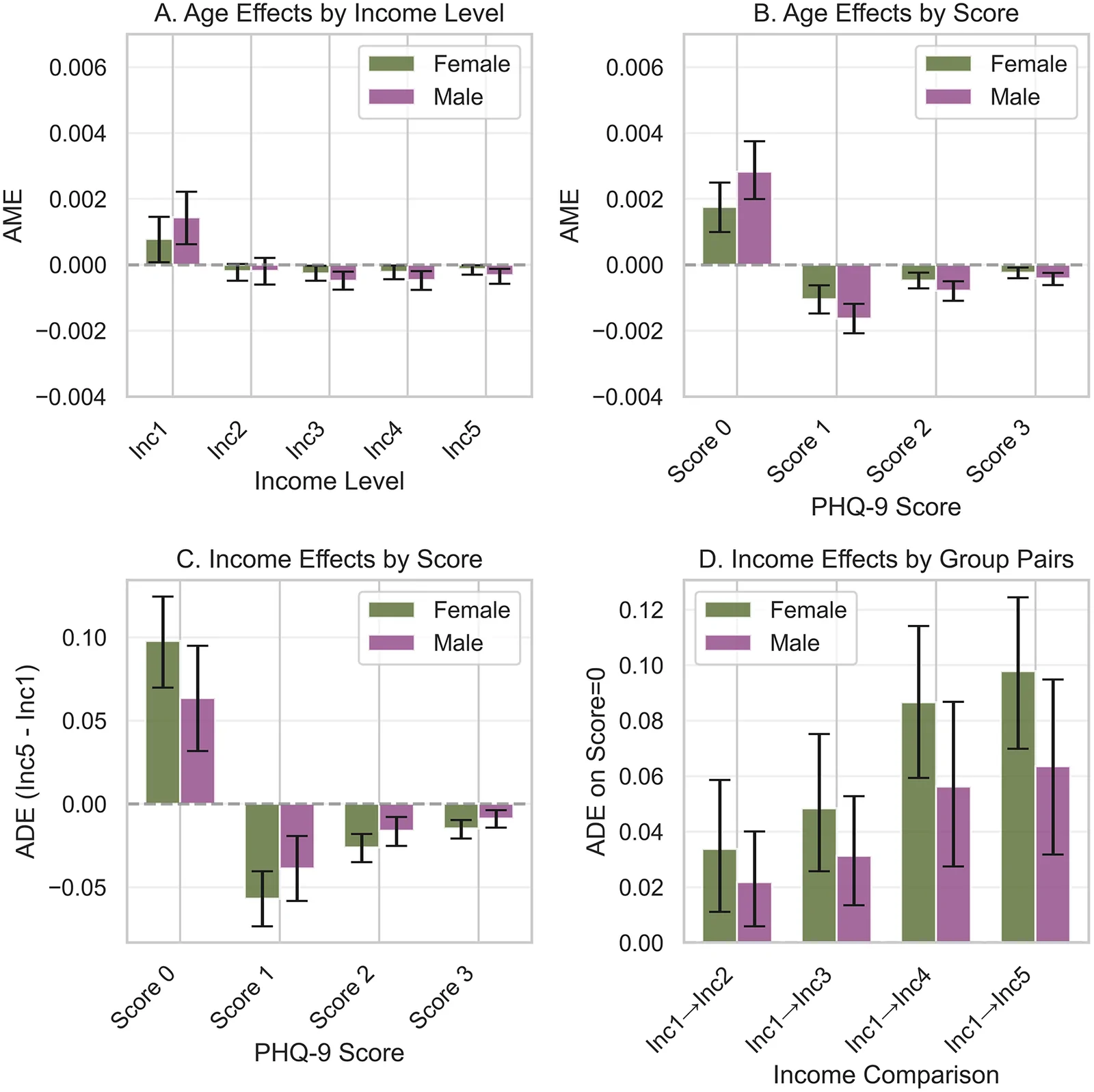
Summary of effects on PHQ-9 scores during Wave-6. (A) Effects of age on weekly household income levels; effects are presented as average marginal effects (AMEs) for males and females across the five income levels; each AME value represents unit percentage point change in income by year of age (20 years old minimum and 84 years old maximum). (B) Age AMEs on PHQ-9 four possible scores [“Not at all (0)”, “Several days (1)”, “More than half the days (2)”, “Nearly every day (3)”]; as before, AME represents the unit change in percentage by 1-year age increase. (C) Effects of income on PHQ-9 scores are expressed as average discrete effects (ADEs), which represent the ordered category probability difference between the lowest income group (Inc1 (£0-£300)) to the highest (Inc5 (£1,112+)) (i.e., Inc5 probability - Inc1 probability). (D) Effects of income on scoring 0 (“Not at all”) on any PHQ-9 item are expressed as ADEs, x-axis shows every increase from Inc1 (lowest income group) to every other income level.

Fig 3 illustrates the disaggregated results to show the change by income level and detail differences with respect to Wave-1. As expected, in Wave-6, age effects on income remained relatively constant with respect to Wave-1 (Fig 3A). For each one-year increase in age, the probability of scoring 0 on PHQ-9 items increased and the probability of answering any other score decreased, with little difference between males and females (Fig 3B). However, when comparing the full age range (e.g., 18–83 years), the cumulative age effects differed substantially between genders (Table 2). Income effects on the probability of scoring 0 on PHQ-9 items were similar in Wave-6 and Wave-1 for males. For females, however, the highest income group had a higher probability of scoring 0 than the lowest income group in Wave-6, reversing the pattern observed in Wave-1 (Figs 2C and 3C). These income effects were larger for females than for males, and this pattern held across all income groups when compared to the lowest income group (Fig 3D).

**Fig 4:**
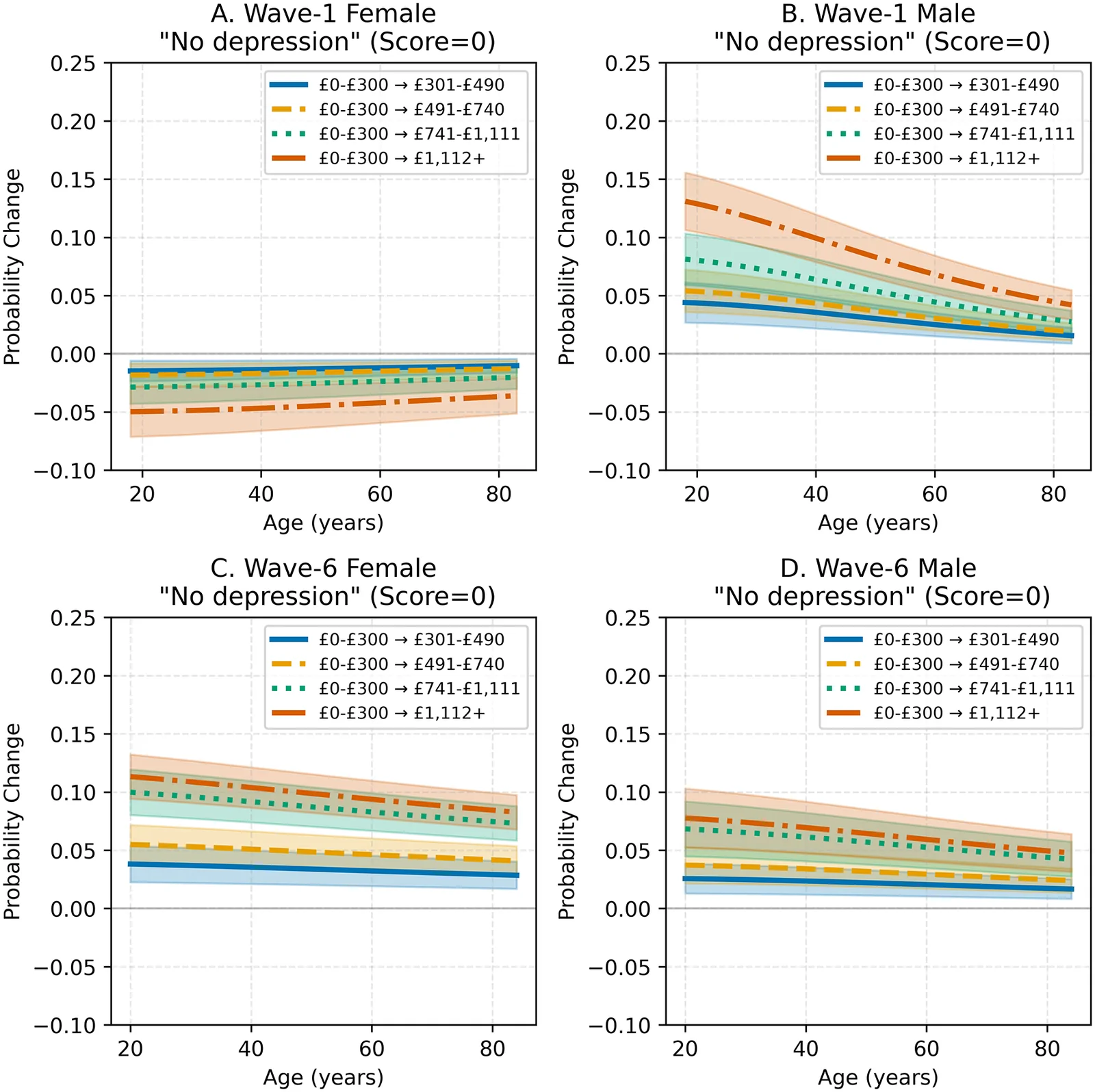
PHQ-9 average partial effect (APE) curves. Lines indicate posterior means and shaded areas show posterior standard deviations. APE values correspond to the expected difference between the probability of scoring 0 (“Not at all”) on PHQ-9 items for the lowest income level (Inc1 = £0-£300) to every other income level across age in years old (higher income groups - the lowest income group).

Fig 4 summarises average partial effects (APEs) of income on the probability of scoring 0 on PHQ-9 items across the sampled age. Positive APEs indicated that higher income groups have higher probability of scoring 0 on PHQ-9 items than lower income groups. Such income effect was stronger in younger population than in older population, and it was stronger for all sampled age for females but weaker for males in Wave-6 relative to Wave-1. The APEs are bigger in males than in females across age, but such gender difference is smaller in Wave-6 than in Wave-1. Fig 4 also indicates that the lowest probability of scoring 0 happened for the lowest income level and progressively increased up to the highest income level.

### 2.2 Anxiety results

**Fig 5:**
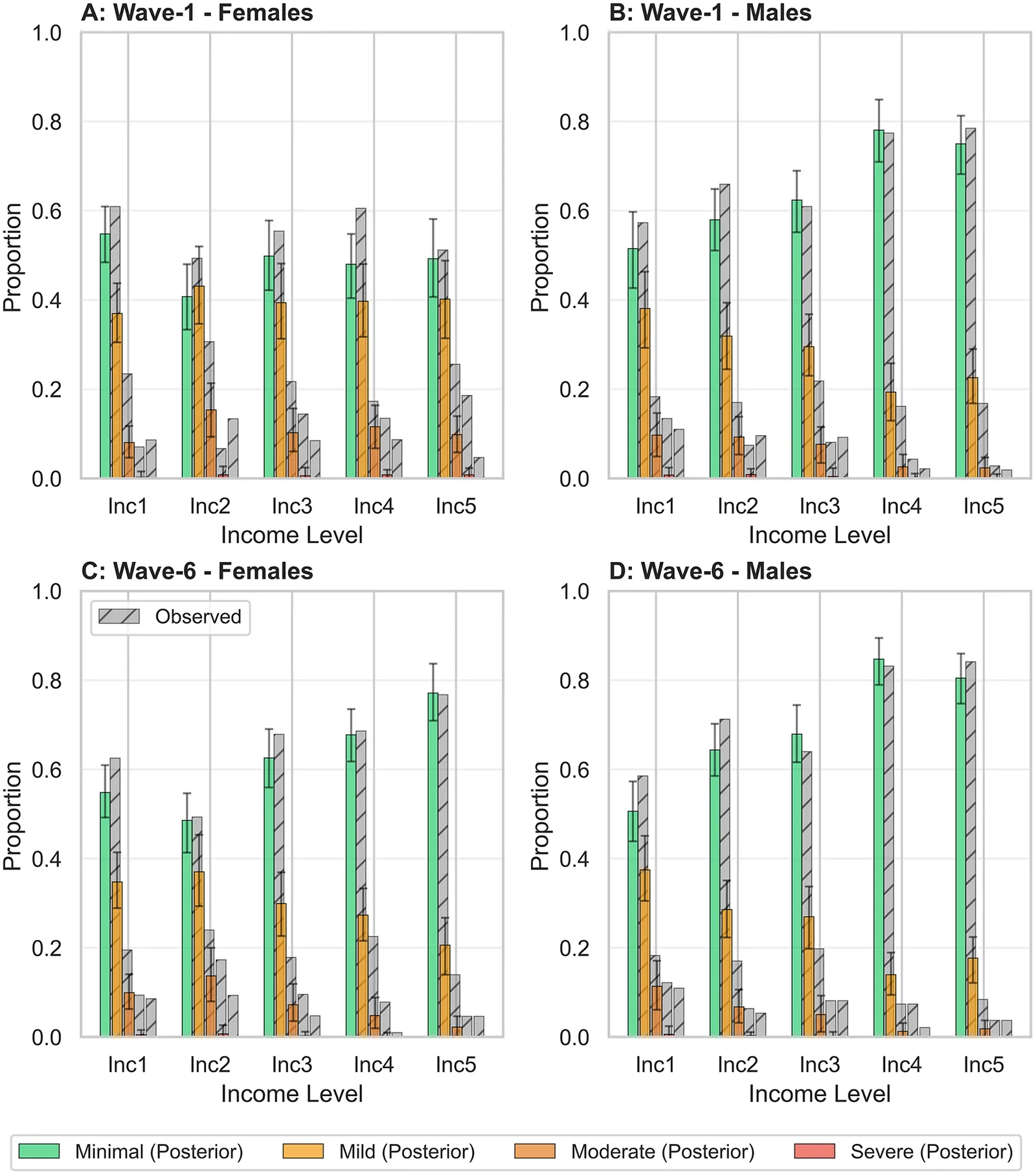
Posterior predictive distributions of GAD-7 scores by income and anxiety severity. Bars represent posterior predictive means; error bar represent 90% HDIs (highest density intervals). Hatched bars show the observed proportion of responses within each GAD-7 threshold.

Wave-1 hierarchical Bayesian mediation models showed that males in income groups 2–5 had a higher estimated probability of scoring below the GAD-7 minimal-anxiety threshold than females. Among males, this probability increased with income through income groups 1–4 and with a slight decrease in income group 5. In contrast, the probability were similar across income groups in females. In the lowest income group (Inc1), the probabilities for both genders were very similar (males: Inc1 = 52% [44%, 60%], Inc2 = 58% [51%, 65%], Inc3 = 62% [55%, 69%], Inc4 = 78% [72%, 84%], Inc5 = 75% [68%, 81%]; females: Inc1 = 55% [48%, 62%], Inc2 = 41% [33%, 48%], Inc3 = 50% [42%, 57%], Inc4 = 48% [40%, 55%], Inc5 = 49% [41%, 58%]) (Fig 5).

**Fig 6:**
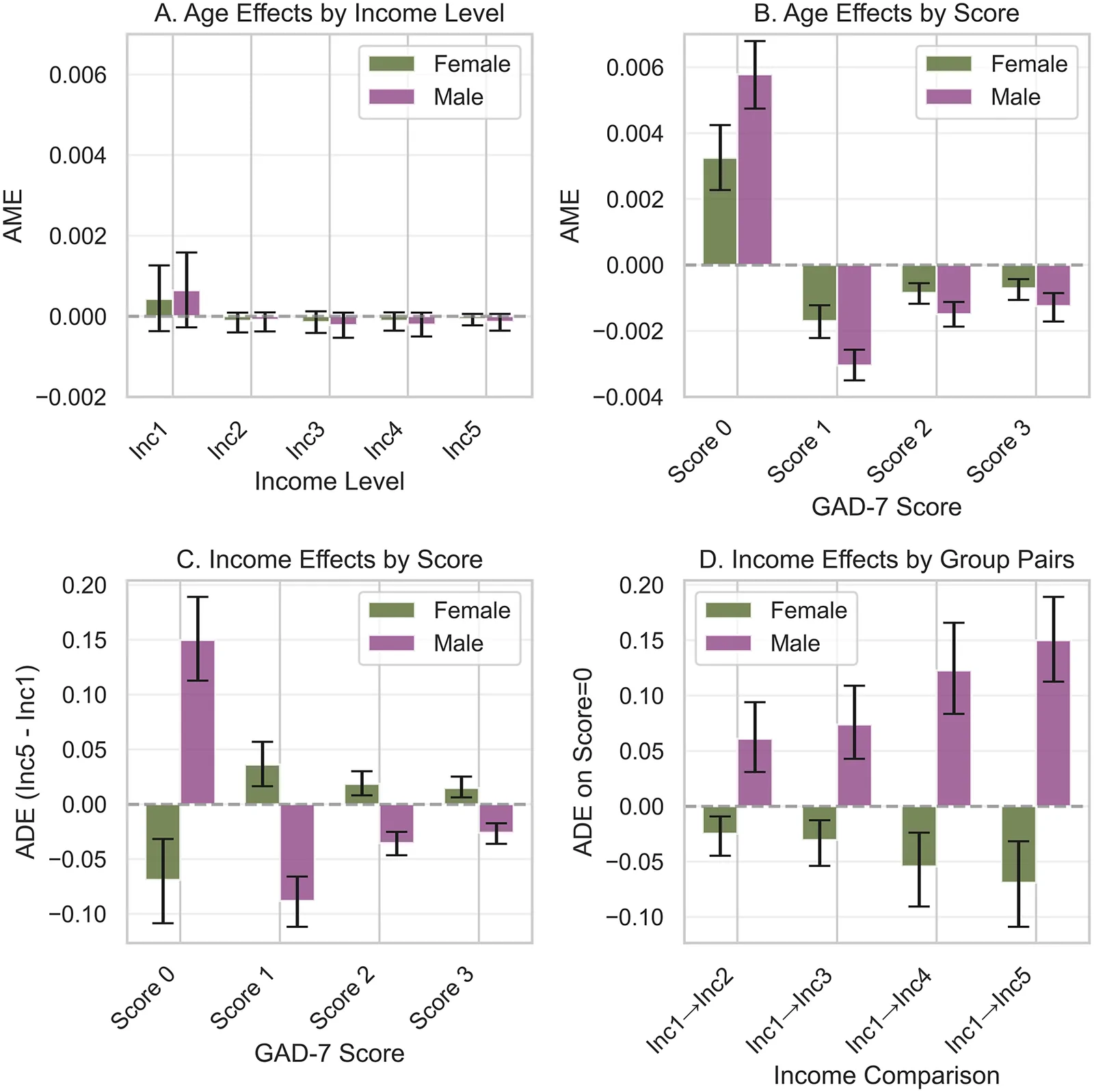
Summary of effects on GAD-7 scores during Wave-1. (A) Effects of age on weekly household income levels; effects are presented as average marginal effects (AMEs) for males and females across the five income levels; each AME value represents unit percentage point change in income by year of age (18 years old minimum and 83 years old maximum). (B) Age AMEs on GAD-7 four possible scores [“Not at all (0)”, “Several days (1)”, “More than half the days (2)”, “Nearly every day (3)”]; as before, AME represents the unit change in percentage by 1-year age increase. (C) Effects of income on GAD-7 scores are expressed as average discrete effects (ADEs), which represent the ordered category probability difference between the lowest income group (Inc1 (£0-£300)) to the highest (Inc5 (£1,112+)) (i.e., Inc5 probability - Inc1 probability). (D) Effects of income on scoring 0 (“Not at all”) on any GAD-7 item are expressed as ADEs, x-axis shows every increase from Inc1 (lowest income group) to every other income level.

For males, the probability of scoring 0 on GAD-7 items was 14.9 percentage points higher in the highest income group than the lowest. By contrast, this income gap was smaller and in the opposite direction for females (–6.7 percentage points; Table 3 and Fig 6C). Additionally, the linear age effect increased the probability of scoring 0 by 33.4 percentage points in males and 21.6 percentage points in females across the sampled age range (Table 3 and Fig 6B). However, the indirect effect of age through income on this probability was negligible.

**Table 3:** Wave-1 summary of effects on GAD-7 (averaged over questions).

Effect	Gender	Mean	SD	5% HDI	95% HDI	Relationship
Mediator	Female	-0.067	0.057	-0.161	0.025	b:Income→Score
Mediator	Male	0.149	0.061	0.049	0.251	b:Income→Score
Direct	Female	0.216	0.127	0.024	0.437	c:Age→Score
Direct	Male	0.334	0.117	0.131	0.515	c:Age→Score
Indirect	Female	0.000	0.001	0.000	0.001	ab:Age→Income→Score
Indirect	Male	-0.001	0.001	-0.003	0.001	ab:Age→Income→Score
Total	Female	0.217	0.127	0.021	0.435	c+ab
Total	Male	0.333	0.117	0.147	0.531	c+ab

Note: In this table, the income effect is defined as the age- and item-averaged probability of answering ‘Not at all’ (score 0) on any GAD-7 question for the highest income group (£1,112+) minus the corresponding probability for the lowest income group (£0–£300). The effect of age corresponds to the increase of the income- and item-averaged probability from the lowest age (18 years old) to the highest age (83 years old). HDI corresponds to the 90% highest density interval.

Age effects on income in different income groups differed negligibly for both genders (Fig 6A). As expected, the probability of scoring 0 on GAD-7 items increased with age, while the probability of scoring 1–3 decreased. This age-related increase was nearly twice as large in males as in females (Fig 6B). The income effects are positive in males whilst negative in females (Fig 6C), and this pattern held across all income groups when compared to the lowest income group (Fig 6D).

In Wave-6, males also presented higher probabilities than females of scoring below the GAD-7 minimal-anxiety threshold across income groups 2–5, with the exception of Inc1, where the probabilities for males were slightly lower than that for females. Among both genders, this probability increased with income generally (males: Inc1 = 51% [44%, 57%], Inc2 = 64% [59%, 70%]], Inc3 = 68% [62%, 74%], Inc4 = 85% [79%, 89%], Inc5 = 80% [75%, 86%]; females: Inc1 = 55% [41%, 61%], Inc2 = 49% [41%, 55%], Inc3 = 63% [56%, 69%], Inc4 = 68% [62%, 74%], Inc5 = 77% [71%, 84%]) (Fig 5).

**Fig 7:**
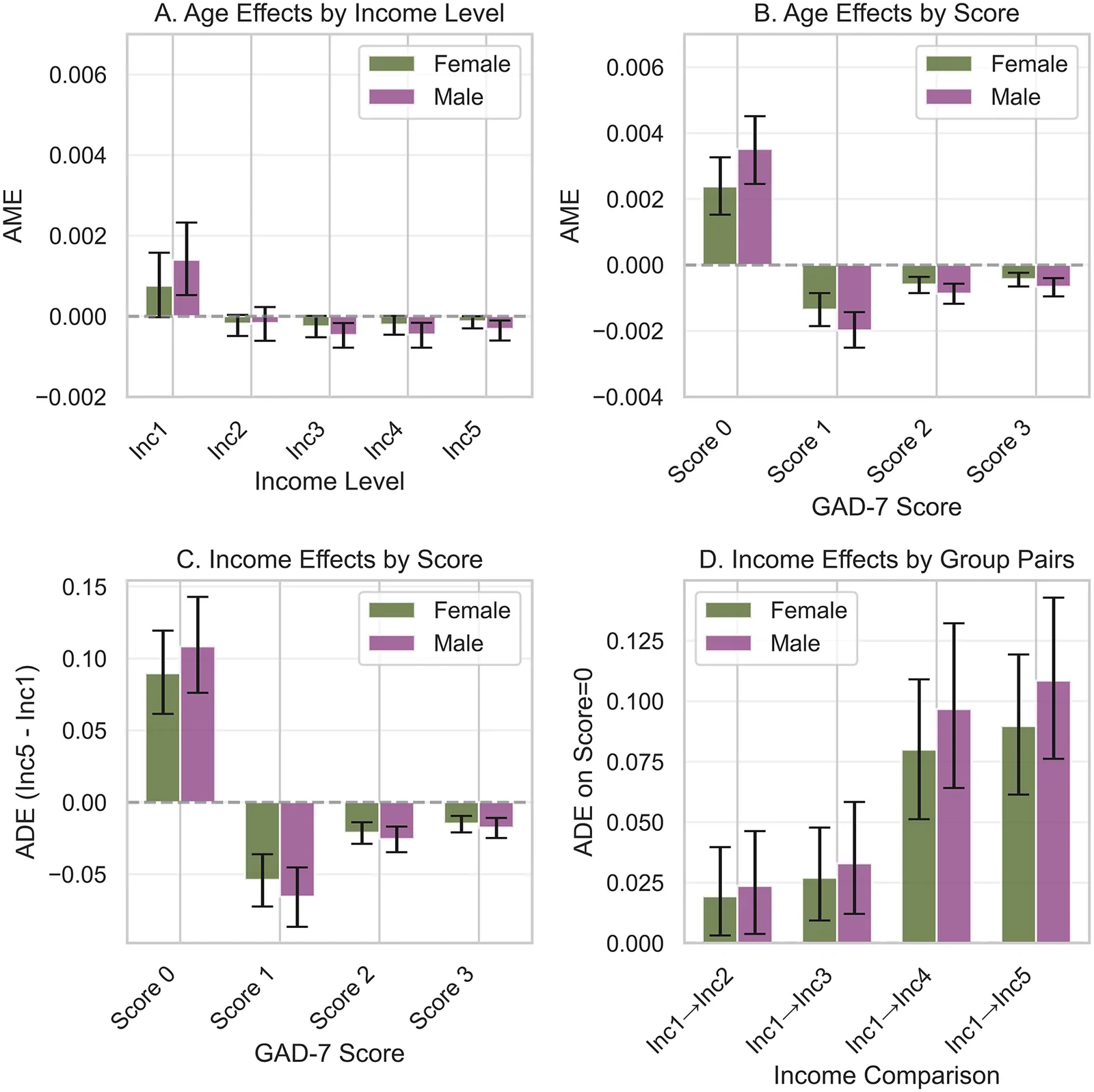
Summary of effects on GAD-7 scores during Wave-6. (A) Effects of age on weekly household income levels; effects are presented as average marginal effects (AMEs) for males and females across the five income levels; each AME value represents unit percentage point change in income by year of age (20 years old minimum and 84 years old maximum). (B) Age AMEs on GAD-7 four possible scores [“Not at all (0)”, “Several days (1)”, “More than half the days (2)”, “Nearly every day (3)”]; as before, AME represents the unit change in percentage by 1-year age increase. (C) Effects of income on GAD-7 scores are expressed as average discrete effects (ADEs), which represent the ordered category probability difference between the lowest income group (Inc1 (£0-£300)) to the highest (Inc5 (£1,112+)) (i.e., Inc5 probability - Inc1 probability). (D) Effects of income on scoring 0 (“Not at all”) on any GAD-7 item are expressed as ADEs, x-axis shows every increase from Inc1 (lowest income group) to every other income level.

Similar to Wave-1, the average probability of scoring 0 on GAD-7 items was higher in the highest income group than the lowest, with a gap of 10.9 percentage points for males and 8.9 percentage points for females (Table 4 and Fig 7B). Age also increased this probability, though more substantially in males (20.2 percentage points) than in females (13.9 percentage points) (Table 4). There was no evidence of a major mediation of age by income in this sample (Table 4). Expanded summaries (by question) can be found in the supporting information of this study (Table C in S1 Appendix for Wave-1, Table D in S1 Appendix for Wave-6).

**Table 4:** Wave-6 summary of effects on GAD-7 (averaged over questions).

Effect	Gender	Mean	SD	5% HDI	95% HDI	Relationship
Mediator	Female	0.089	0.051	0.007	0.174	b:Income→Score
Mediator	Male	0.109	0.066	-0.002	0.217	b:Income→Score
Direct	Female	0.139	0.093	-0.005	0.295	c:Age→Score
Direct	Male	0.202	0.107	0.036	0.380	c:Age→Score
Indirect	Female	-0.001	0.001	-0.002	0.000	ab:Age→Income→Score
Indirect	Male	-0.002	0.002	-0.005	0.000	ab:Age→Income→Score
Total	Female	0.139	0.093	-0.005	0.295	c+ab
Total	Male	0.200	0.107	0.036	0.379	c+ab

Note: In this table, the income effect is defined as the age- and item-averaged probability of answering ‘Not at all’ (score 0) on any GAD-7 question for the highest income group (£1,112+) minus the corresponding probability for the lowest income group (£0–£300). The effect of age corresponds to the increase of the income- and item-averaged probability from the lowest age (20 years old) to the highest age (84 years old). HDI corresponds to the 90% highest density interval.

Fig 7 provided disaggregated effects of age and income. As expected, age effects on income remained relatively constant with respect to Wave-1 (Figs 6A and 7A). As age increased, the probability of scoring 0 on GAD-7 items increased and that of the other scores decreased. This age effect was similar across genders (Fig 7B). Unlike Wave-1, the income effects were positive for both genders, although the magnitude was smaller for males than previously estimated. This pattern was consistent across all income groups when compared with the lowest group (Fig 7C and 7D).

**Fig 8:**
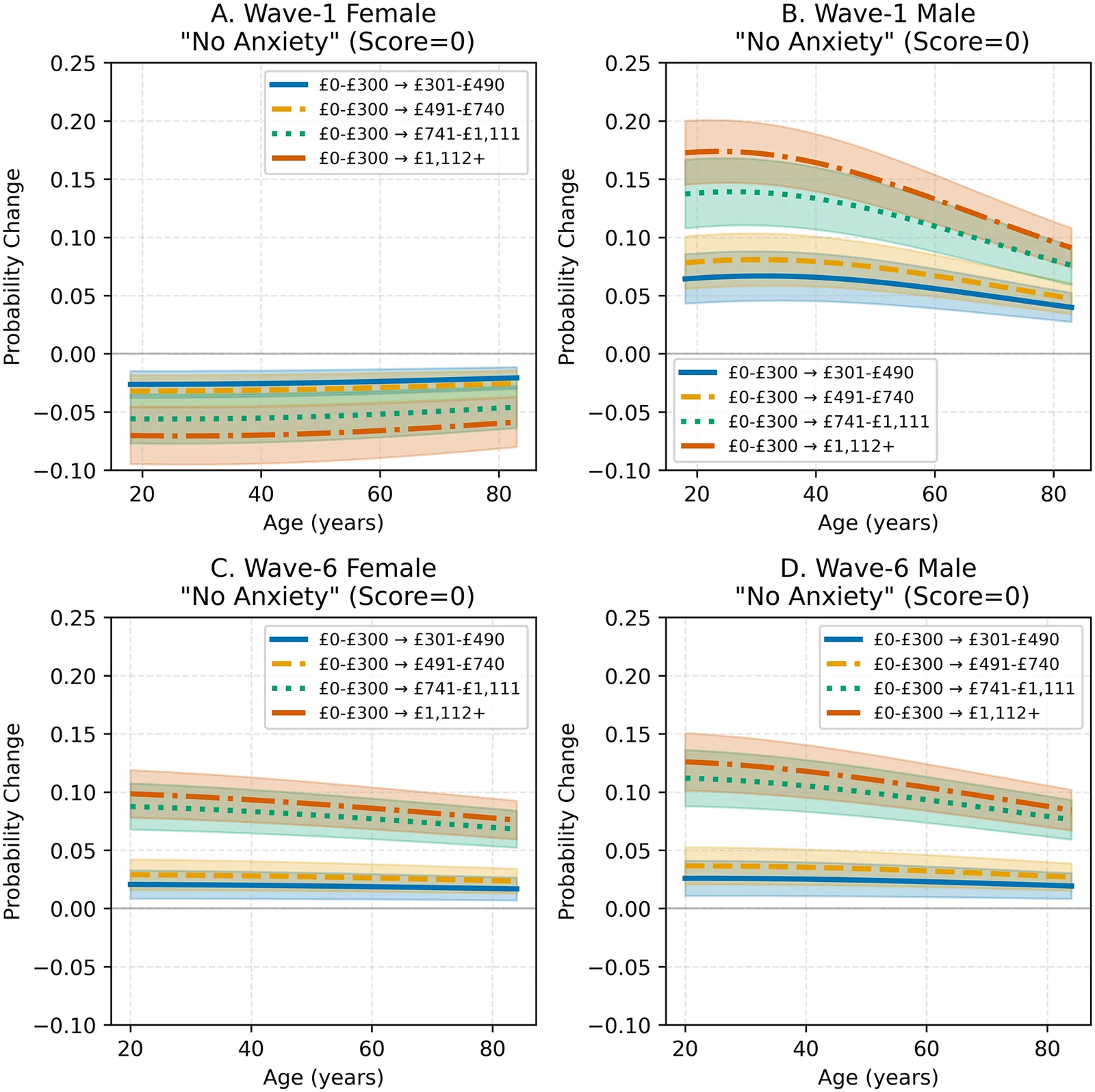
GAD-7 average partial effect (APE) curves. Lines indicate posterior means and shaded areas show posterior standard deviations. APE values correspond to the expected increase in percent points for scoring 0 (“Not at all”) in GAD-7 from the lowest income group to every other income group across age in years old.

Fig 8 summarises income APEs on scoring 0 on GAD-7 times across the sampled age. For males, higher income groups have a greater probability of scoring 0 on GAD-7 items across all ages, with this effect being more pronounced among younger individuals. For females, income had a negative effect on this probability across all ages in Wave-1, but the effect across income groups were similar in magnitude. In Wave-6, the income effect among females became positive, similar to males. This income effect was also more pronounced at younger ages than at older ages, with the exception of Wave-1 females, for whom the age modulation was negligible.

### 2.3 Summary of results

For males across all income groups, and for females in the lower income groups (Inc1, Inc2 and Inc3), the probability of falling into the minimal-or-none depression category was lower at Wave-6 than at Wave-1. In contrast, for females in the higher income groups (Inc4 and Inc5), this probability increased over time (Fig 1). In both waves, higher-income males were more likely to fall into the minimal-or-none depression category and to score 0 on individual PHQ-9 items. Among females, this same pattern appeared only at Wave-6 (Figs 1, 2C and 3C). At Wave-1, however, the female pattern differed. Those in Income group 2 had a substantially lower probability of minimal-or-none depression compared to the other income groups, while the remaining groups showed similar probabilities to one another (Fig 1), despite that females with more income had lower probability of scoring 0 on individual PHQ-9 items (Table 1 and Fig 2C). Interestingly, in Wave-6, the female income-depression relationship, for both overall category and individual item scores, demonstrated the same pattern as that estimated in males (Figs 1 and 3 and Table 2).

Both genders had higher probability of falling into the ‘minimal or none’ anxiety category at Wave-6 than at Wave-1 across Income groups 2–5. In contrast, this probability of the lowest income group for both genders stayed constant. Also, in both waves, this probability for males was generally higher than that for females (Fig 5). Among males, higher income was linked to a lower probability of endorsing any anxiety symptoms (i.e., scoring above 0 on individual GAD-7 items) in both waves (Tables 3 and 4 and Figs 6C and 7C). However, this income effect weakened from Wave-1 to Wave-6 (Tables 3 and 4). Among females, the pattern was more complex. In Wave-1, higher-income females showed a slightly lower probability of scoring 0 on individual GAD-7 items (Table 3 and Fig 6C). However, the probability of falling into the minimal-or-none overall category was similar across most female income groups, with the exception of Inc2, which showed a noticeably lower probability (Fig 5). By Wave-6, this relationship reversed for females: higher income predicted a greater probability of scoring 0 on individual items and, correspondingly, a higher probability of falling into the ‘minimal or none’ overall anxiety category in general (Table 4 and Figs 5 and 7C).

In summary, the model’s estimated age effect on income was stable across waves, as expected given the nearly one-year age progression for all participants. Additionally, results from the observed sample showed no evidence of income-mediating age effects on either anxiety or depression measures, as evidenced by negligible indirect effects from the model. Posterior predictive checks and additional figures summarising the income and age effects by question can be found in the supporting information of this study (Fig A-N in S2 Appendix).

## 3. Discussion

Our research examined how age and income jointly influence mental health across genders and age groups, using data from two waves of the C19PRC study [13], collected in March 2020 (Wave-1) and August–September 2021 (Wave-6), during the COVID-19 pandemic in the UK. A causal model via a Bayesian hierarchical ordered-logistic regression was used for statistical analyses, revealing relevant effects of income on PHQ-9 (depression symptoms scale) and GAD-7 (generalised anxiety disorder scale) measures. The probabilities of scoring 0 on anxiety and depression questionnaire items increased as a function of both age and income, with a non-linear relationship between income and age. These effects also presented some gender differences. Age had a stronger effect on depression and anxiety for males than females in both waves. Income also had a larger protective effect against anxiety for males in both waves. For depression, income followed the same male-larger pattern at Wave-1, but this reversed by Wave-6, with females showing a larger income effect.

The relationship between income and depression/anxiety levels also showed gender differences. For depression, the estimated probability of minimal-or-none depression decreased from Wave-1 to Wave-6 for males across all income groups and for females in the lower income groups, but this decrease was smaller in the higher income groups for both genders, and reversed to an increase for females in the top two income groups. Also, at both waves, males had higher estimated probabilities than females in the same income groups, with two exceptions: the lowest income group (at both waves) and the highest income group (at Wave-6 only), where the probabilities were similar for both genders. For anxiety, both genders showed a higher probability of minimal-or-none anxiety at Wave-6 than Wave-1 across most income groups, except the lowest. For males, the increase was similar across income groups. For females, the increase was larger than for males in all income groups except the lowest, and the magnitude increased with income. Nonetheless, this probability was higher in males than in females in the same income groups at both waves.

### 3.1 Income, mental health, and gender differences

Consistent with prior literature (e.g., [1,23]), our results indicate that higher income is associated with lower probability of getting depression and anxiety. This reinforces the role of income as a critical determinant of mental health [21,24]. However, our results partially contrast with some of this literature. For instance, Ettman and colleagues [4] investigated the influence of annual household income, accrued financial assets (savings), and other financial factors on mental health (measured with GAD-7 and PHQ-9) during the COVID-19 pandemic from 2020-2023 in the United States. Their research found that savings, but not annual household income, was associated with symptoms of anxiety. Also, they did not find evidence of relevant influence of their main factors on depression.

This discrepancy may reflect contextual factors: our UK-based data align with previous studies conducted in European countries, which demonstrated that income has a stronger association with mental health than wealth. The stronger association between income (as opposed to accumulated wealth) and mental health may be attributed to the more generous European social support systems [25], which buffer individuals from sudden large expenses, such as emergency medical treatment. Wealth (e.g., savings or accrued assets) typically enables individuals to cover large, one-off expenses, such as COVID-19-related medical bills, whereas income primarily sustains recurring expenses like rent, utilities, and groceries. This distinction is critical in the pandemic context, where both sudden financial shocks (e.g., hospitalization) and sustained economic strain (e.g., job loss) exacerbated mental health risks. Consequently, the relative importance of income versus wealth for mental health likely varies across national contexts, depending on the strength of social safety nets and the structure of healthcare systems. Our findings provide evidence that income is a robust predictor of mental health in the UK during the COVID-19 pandemic. The significant strengthening of this relationship from Wave-1 to Wave-6 suggests that financial resources became progressively more important for preserving mental well-being, potentially by enhancing resilience against prolonged socioeconomic disruption.

Recent literature on income and mental health has observed a higher incidence of depressive symptoms on females during pre-pandemic times [19,20]. Consistent with these findings, our results indicate that females’ probabilities of having minimal-or-none depression or anxiety almost consistently remained below males’ during the pandemic too. Also, females mental health probability showed a non-linear pattern, with the second-lowest income group showing the poorest mental health. This may reflect a cliff effect in pandemic support eligibility, whereby females in this group earned too much to qualify for government financial assistance or social housing, yet their income was reduced (e.g., through the CJRS furlough scheme) to a level that left them unable to afford buffers (e.g., childcare, delivery services) available to higher-income peers [26]. Another possibility is that females from this income level may have been in precarious, non-essential jobs or work-from-home jobs with intense pressure (e.g., call centres, admin) while also needs to manage homeschooling and domestic duties. Thus, females from the second-lowest income group are uniquely exposed to pandemic-related gender-specific stressors.

The Bayesian hierarchical ordered logistic regression revealed a marked sex difference at the beginning of the pandemic (Wave-1). Higher income was associated with lower levels of depression and anxiety among males, whereas no such association was observed among females, either in the likelihood of reporting no symptoms on the GAD-7 and PHQ-9 or in overall symptom severity. This finding suggests that the mental health benefits typically associated with higher income were less pronounced for women during the initial phase of the pandemic. A possible reason is that lockdown measures curtailed access to services and activities that financial resources would ordinarily facilitate, while simultaneously increasing caregiving and household responsibilities. Under these conditions, income may have been less effective in mitigating psychological distress among women than among men.

In contrast, the fact that income has a constantly strong influence on mental health in males is likely due to sociocultural norms positioning men as primary earners, for whom income may more strongly symbolize status and success [27,28]. This may also explain why, despite males generally having a higher probability of minimal-or-none depression and anxiety than females, those in the lowest income groups had similar probabilities to their female counterparts and appeared to be equally affected by the pandemic.

By Wave-6, however, although the income protective effect on both mental health issues had decreased slightly in males, it increased greatly in females. At this stage, although the effect was still stronger in males than in females on anxiety, it was stronger in females on depression. This suggests that females are not only more vulnerable, in terms of mental health, to a pandemic like COVID-19 but are also more generally susceptible to the influence of socioeconomic stress/alleviation across the life course. This feature may reflect previous findings, which indicate that females tended to have more responsibility than males within the household during the COVID-19 pandemic as the stay-at-home time of household members increased [17]. Women were found to be disproportionately burdened by depression with respect to men, and low-income pregnant and parenting women have particularly high rates of depression and often lack access to treatment [29]. In contrast, women in higher-income households had greater access to external support services (e.g., grocery delivery, cleaning, and dining out) to offset domestic burdens, which was an advantage that became more pronounced as lockdown measures were gradually lifted. Taken together, these findings underscore the critical role of income in shaping mental health outcomes and females’ relative vulnerability during the pandemic.

### 3.2 Age, mental health, and gender differences

Age also plays an important role in mental health, but the effect of age on wellbeing and mental health is controversial. While some studies, particularly in economics, report a U-shaped association between age and subjective wellbeing, encompassing both life satisfaction and short-term emotional states [30], the robustness of this pattern appears contingent on model specification. Blanchflower and Oswald [31] demonstrate that this U-shaped relationship only emerges when conditioning on potential confounders such as income. Conversely, Frijters and Beatton [30] argue that the U-shaped profile becomes statistically significant only after incorporating standard socioeconomic covariates. These contradictory findings suggest that the age-wellbeing relationship may be highly sensitive to analytical approaches and variable selection. This highlights the relevance of designing clear causal models which specify appropriate relationships between variables.

Recent large-scale research employing causal models has documented a positive relationship between income and wellbeing. For example, Li and Managi [21] analysed a global dataset comprising approximately 1.6 million observations across 163 countries from 2009 to 2022, examining how age and income influence wellbeing. Their findings revealed that while advancing age consistently predicted higher income, it was simultaneously associated with lower levels of wellbeing, even though income conferred only marginal gains in wellbeing at higher ages. However, this relationship may vary significantly across national contexts [31] and temporal periods, particularly during major disruptions such as the COVID-19 pandemic and economic contractions [32]. Importantly, Li and Managi [21] measured wellbeing solely as general life satisfaction (a notion of subjective wellbeing, for details see: [33]. When wellbeing is instead operationalised using specific emotional states, age-related patterns differ. Stone et al. [34] examined multiple wellbeing dimensions in adults aged 18–85, including stress, anger, and worry; the core feature of GAD and directly relevant to our study’s measures. Their results demonstrated that these measures generally decreased linearly with age, contrasting with the life satisfaction findings.

In contrast to previous research [21], participants in our research reported their yearly household income from 2019, which was used for both waves. Age increased uniformly by one year for all participants, limiting meaningful variation in the age-income relationship. As a result, the minimal association between age and income could be artefactual. Accordingly, the direct effect of age on income was examined as a relevant secondary exposure. This research focuses more on the joint influence of age and income, together with gender, on mental health, and how the effect of income on mental health varies across age.

Another difference between our study and previous ones is that we define mental health specifically through the lens of GAD and MDD. While anxiety disorders encompass multiple conditions (e.g., panic disorder, social anxiety), we specifically focus on GAD, characterised by its core feature of persistent worry [35]. GAD is primarily linked to anxious apprehension [36], manifesting as chronic worry, rumination, and future-oriented threat processing [37]. Notably, anxiety symptoms often co-occur with depressive disorders due to shared underlying mechanisms [38].

We assessed GAD and MDD using the GAD-7 and PHQ-9 questionnaires respectively, which are validated instruments widely employed in clinical and research settings [39]. Our Bayesian hierarchical ordered-logistic regression results indicated a decline in risk of anxiety and depression symptoms as age increases. We note, however, this protective age effect on mental health was smaller in Wave-6 than in Wave-1. This pattern may be due to COVID-19 itself: greater susceptibility of the COVID-19 virus among middle-aged and older populations may have induced heightened levels of distress in older age groups.

In addition, age effect on mental health on females were constantly smaller than that on males. This may be due to the traditional social roles. Our findings suggest that the attenuated protective effect of age among females is not due to an absence of resilience in older women, but rather to the simultaneous and intensifying activation of traditional caregiving roles that accompany female aging. While older women exhibit greater emotional regulation and crisis-coping skills than their younger counterparts, these protective resources are systematically offset by escalating exposures to intrahousehold care work and health surveillance of family members. In contrast, older males experienced the protective benefits of age without a commensurate increase in these role-based stressors, resulting in a stronger positive age effect on mental health.

Moreover, although income does not meaningfully mediate the age-mental health relationship, the strength of the association between income and mental health differs across age. In our study, the influence of income on mental health was smaller in older adults relative to younger adults. This is consistent with the result from an earlier review, which documented reduced risk of the occurrence of such disorders across the adult life span [22]. The review attributed this pattern to decreased emotional responsiveness, increased emotional control and psychological immunisation to stressful experiences developed with age. Our results may have provided evidence for these mechanisms, suggesting that age-related resilience may reduce the effect of income on mental health. Interestingly, although this pattern has been observed for both genders and across waves, it was primarily among males during the early pandemic. This gendered difference likely reflects what income symbolically represents as discussed above: for males, high income signals status and success, a function diminished by pandemic-related social isolation; for females, income is usually related to relief from intrahousehold work and childcare, especially for younger females with dependent children. Therefore, while income’s protective role weakened with age for males, it persisted for females, particularly younger females, as its utility is tied to practical domestic support rather than external validation. Future research could explore this phenomenon on other relevant factors, such as wealth, safety nets, social welfare and/or protection, or similar variables.

### 3.3 Limitation

It is important to note that our study included only participants aged 18 years or older. According to the World Health Organization’s definition, pre-adulthood spans from 0 to 19 years old. Also, non-white ethnicities were excluded from the sample due to their insufficient representation (6.94% of the total participants). This means our sample only encompasses adults (young, middle age, and older adults) and UK population of white ethnicity. Thus, the presently observed linear relationship between income and mental health may not generalise to other age or ethnic groups, such as childhood (0–9 years old), adolescence (10–19 years old), or any UK population of non-white ethnicities. For instance, family income may be associated with different developmental factors during the childhood-adolescence transition, which could lead to non-linear changes across various mental health measures (e.g., [40]. Future research should incorporate non-adult populations and non-white ethnicities to elucidate how income influences mental health across the lifespan in different ethnicity groups during pandemics. Other relevant avenue of research could be investigating gender differences broadly, beyond female-male differences.

In addition, our study used relatively balanced samples across age and income groups (a control measure implemented by C19PRC researchers). However, relevant for our present purposes, this approach does not reflect the actual demographic distribution observed in real-world populations. In most regions, the proportion of individuals within each age and income stratum varies, with very high-income groups typically constituting a smaller subset compared to other income categories—a pattern similarly observed in age distributions. By such similar group sizes, our study inadvertently overrepresented high-income individuals, thereby amplifying their influence on the results. Consequently, the observed effects of income differences may be attenuated compared to real-world scenarios. Future research could consider adjusting sample sizes to align with the true demographic proportions of the target population, ensuring more generalisable and contextually relevant findings.

### 3.4 Implications and future directions

The evidence provided from our methodological approach can provide useful information for understanding the current dynamics of age, gender and income during public emergencies. In time, these can be relevant to inform targeted mental health policies and economic support programs for future emergencies. Given the very specific focus of our study, we could not explore impact of policies such as the self-employed income support scheme (SEISS) or the furlough scheme, intended to distribute financial support to employees [41]. Having adjusted for variables such as employment, home ownership, education, or having children, we observed that gender differences in relation to household income and mental health persist. Considering previously observed gender disparities in this respect [19,20], direct distribution policies may require re-enforcement, differentiation (e.g., sex or gender-based), and or supplement depending upon household composition to acknowledge gender differentiated effects. Other policies, perhaps not targeting income directly (e.g., food basket supplementation, care assistance) could also help to alleviate the impact of income on mental health by increasing disposable income, increasing saving capacity, or similar beneficial effects.

Given the scope and limitations of our study, there are several gaps and improvements which could be addressed in future research. First, our focus was on exploring a very specific set of the C19PRC sample, such as including only participants who took part in the initial wave and the sixth, so we could have the best comparability of responses possible across participants. However, C19PRC has a panel structure, which includes refreshment samples. Future studies focusing on broader population level patterns could take advantage of each measurement wave with models designed to account for the potential bias and heterogeneity induced by wave-to-wave and participant (refreshment) variation. Our study has focused on three main variables (income, gender, and age) given our goals and theoretical concerns. However, there are important relationships to explore in the context of the pandemic, and other crisis periods, regarding relevant variables such as employment, housing, loneliness, or trauma and their associations with mental health. In short, future research has wide avenues to explore in terms of modelling approaches, influence of different factors, or the combination of both.

### 3.5 Conclusion

In conclusion, this study employed causal Bayesian hierarchical ordered-logistic models to examine the relationships between age, income, and mental health across genders at two distinct stages of the COVID-19 pandemic. Our findings demonstrate that older age was associated with better mental health outcomes, with income exerting a generally independent (and stronger) influence on GAD and MDD. While the age-related mental health trends were consistent between genders, income exerted differential effects on men and women throughout the pandemic. Notably, women exhibited greater vulnerability to mental health challenges than men across most income groups; however, in the lowest income bracket, the gender gap disappeared as both men and women reported similarly elevated levels of distress, highlighting the protective function of income. These results underscore the importance of incorporating demographic and socioeconomic factors into the design of mental health policies and economic support programs during public health emergencies and to focus on the importance which material conditions have for mental health.

## 4. Materials and methods

### 4.1 Data

The data used in the current study is from the COVID-19 Psychological Research Consortium (**C19PRC**) study. The data are publicly available and were accessed on 25 July 2025 from https://sheffield.ac.uk/psychology-consortium-covid19/publications. All data in the database are fully anonymised, and thus, no identifiable personal information was available to the researchers. Although the C19PRC study involved several waves of measurement from 2020 to 2021, we are interested in Wave-1 (March 2020) to Wave-6 (August–September 2021), as their measurements are the most detailed (publicly available) and capture the first two core years of the COVID-19 pandemic in the UK. We use mediation models to identify the effects of age and income, across gender (female and male) on PHQ-9 and GAD-7 scores on Wave-1 and Wave-6 only, as they reflect the beginning and end of measurements.

As we were interested on the influence of COVID-19 pandemic on mental health, we kept only participants who took part in Wave-1 and retook the test during Wave-6 (n = 1100); we excluded participants with unreliable reports of age (n = 89), namely participants declaring being less than one year older or more than two years older in Wave-6 with respect to Wave-1. Participants of non-white ethnicities did not have sufficient numeric representation (n = 65) and were not included. Additionally, five participants who reported inconsistent ethnicities in Wave-6 and two who reported neither female nor male were excluded.

After exclusions, the total number of eligible participants was 939. Participants’ report of their income (defined as total weekly household income in 2019, retrospectively reported in both Wave-1 and Wave-6) were consistent between the two waves, thus no further exclusion was made. Three participants changed gender identification across waves (two male-to-female, one female-to-male) and were retained, as such changes are plausible. The participants were grouped into five weekly income levels: £0 - £300 (n = 210), £301 - £490 (n = 197), £491 - £740 (n = 193), £741 - £1,111 (n = 170), £1,112 or more (n = 169). In Wave-1, participants (males = 476, females = 463) aged from 18 to 83 years old (Mean = 50.28, SD = 15). In Wave-6, participants’ (males = 475, females = 464) age ranged from 20 to 84 years old (Mean = 51.72, SD = 15.01). Dependent variables (outcome variables) were PHQ-9 scores (Wave-1: mean total score = 4.31, SD = 5.51; Wave-6: mean total score = 4.68, SD = 5.84) and GAD-7 scores (Wave-1: mean total score = 4.48, SD = 5.43; Wave-6: mean total score = 3.75, SD = 5.21). We also identified the conventional thresholds for PHQ-9 scores that indicated depression levels as “minimal or none” (0–4, n = 628), “mild” (5–9, n = 167), “moderate” (10–14, n = 75), “moderately severe” (15–19, n = 46) and “severe” (20–27, n = 23). Anxiety levels were identified by GAD-7 scores as “minimal” (0–4, n = 586), “mild” (5–9, n = 194), “moderate” (10–14, n = 88) and “severe” (15–21, n = 71).

### 4.2 Analysis

**Fig 9:**
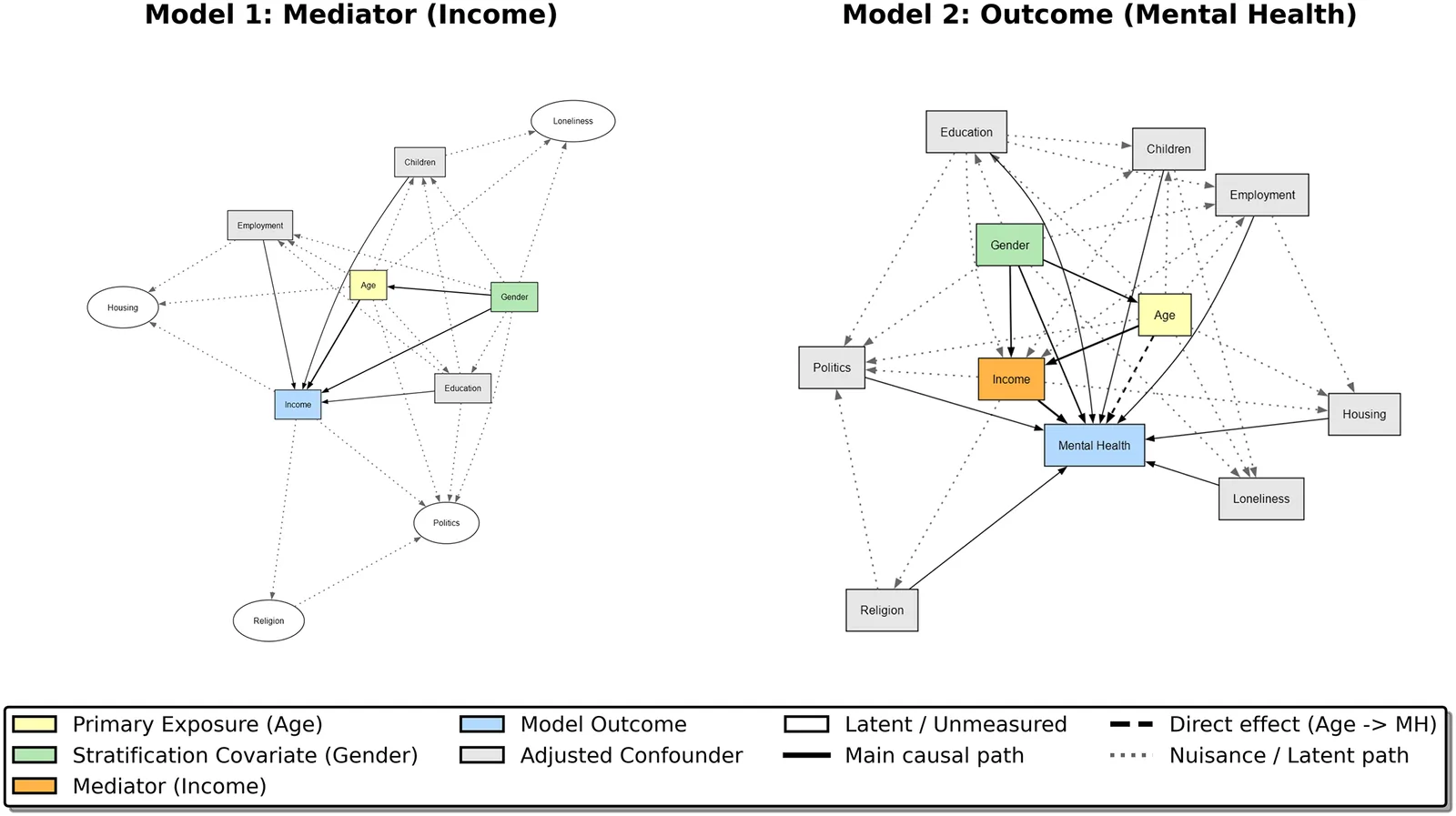
Mediation model framework. The model determines the direct effects of age and income (mediator), stratified by sex, on mental health. Model 1: Sub-model for calculating the direct effects of age and sex on income. Model 2: Sub-model for calculating the direct effects of age, sex and income on mental health measures (PHQ-9 or GAD-7 scores).

To understand the effect of income on mental health outcomes (i.e., depression measured by PHQ-9 and anxiety measured by GAD-7), we estimated a mediation model implemented as a hierarchical ordered-logistic regression. In this framework, age is the predictor, weekly household income is the mediating variable, and mental health item responses are the outcomes (Fig 9). The model allows age to affect mental health both directly and indirectly through income.

#### Mediation model.


$$\begin{array}{c} Mediator: \end{array}$$

$$\begin{array}{c} w_{i}OrderedLogistic\left(\theta_{i},\kappa_{\left(j-\text{1}\right)}\right) \end{array}$$

$$\begin{array}{c} \theta_{i}=\alpha_{\text{1}\left(g\right)}+a_{g}\times age_{z(i)}+U_{\text{1}}^{T}X_{\text{1}\left(i\right)} \end{array}$$

$$\begin{array}{c} \kappa_{j}Normal\left(\left[\text{0}.\text{25},\text{0}.\text{5},\text{1},\text{2}\right],\text{0}.\text{5}\right),ordered \end{array}$$

$$\begin{array}{c} Outcome: \end{array}$$

$$\begin{array}{c} y_{i}OrderedLogistic\left(\eta_{i},\kappa_{\left(q,k-\text{1}\right)}\right) \end{array}$$

$$\begin{array}{c} \eta_{i}=\alpha_{\text{2}\left(q,g\right)}+c_{\left(q,g\right)}\times age_{z\left(i\right)}+b_{\left(q,g\right)}\times \sum_{\left(j=\text{1}\right)}^{\left(W_{i}-\text{1}\right)}\delta_{j}+U_{\text{2}}^{T}X_{\text{2}\left(i\right)} \end{array}$$

$$\begin{array}{c} \kappa_{\left(q,k\right)}Normal\left(\left[\text{1},\text{2},\text{3}\right],\text{0}.\text{5}\right)\;,ordered \end{array}$$

$$\begin{array}{c} Coefficients: \end{array}$$

$$\begin{array}{c} \alpha_{\text{1}\left(g\right)}Normal\left(\text{0},\text{1}\right)\;,g=\text{1},\text{2} \end{array}$$

$$\begin{array}{c} \alpha_{\text{2}\left(q,g\right)}Normal\left(\text{0},\text{1}\right),q=\text{1}..\text{9},g=\text{1},\text{2} \end{array}$$

$$\begin{array}{c} a_{g}Normal\left(\text{0},\text{1}\right),g=\text{1},\text{2} \end{array}$$

$$\begin{array}{c} b_{\left(q,g\right)}Normal\left(\text{0},\text{1}\right),q=\text{1}..\text{9},g=\text{1},\text{2} \end{array}$$

$$\begin{array}{c} c_{\left(q,g\right)}Normal\left(\text{0},\text{1}\right),q=\text{1}..\text{9},g=\text{1},\text{2} \end{array}$$

$$\begin{array}{c} Confounders: \end{array}$$

$$\begin{array}{c} U_{\text{1}}Normal\left(\text{0},\text{0}.\text{25}\right) \end{array}$$

$$\begin{array}{c} U_{\text{2}}Normal\left(\text{0},\text{0}.\text{25}\right) \end{array}$$

$$\begin{array}{c} Incomeweig\text{h}ts: \end{array}$$

$$\begin{array}{c} \delta Diric\text{h}let\left({\text{1}}_{\left(j-\text{1}\right)}\right) \end{array}$$

$$\begin{array}{c} \delta_{j}\geq 0,\sum \delta_{j}=1 \end{array}$$

$$\begin{array}{c} \delta_{\text{0}}=\text{0} \end{array}$$

Our mediation model follows a standard mediation structure implemented in an ordered-logistic framework and consists of two linked sub-models: (i) a mediator model for income and (ii) an outcome model for mental health.

**The mediator model: age → income:** Weekly household income is measured as an ordered categorical variable with J=5 levels. The linear predictor for income $\theta_{i}$ is the linear predictor of the effect of age on weekly income, over i participants,


$$\begin{array}{c} \theta_{i}=\alpha_{\text{1}\left(g\right)}+a_{g}\times age_{z(i)}+U_{\text{1}}^{T}X_{\text{1}\left(i\right)} \end{array}$$

Where $U_{\text{1}}^{T}X_{\text{1}\left(i\right)}$ is the minimal adjustment set (education, employment, and children), $\alpha_{\text{1}\left(g\right)}$ is a gender-specific intercept, z denotes the standardised age, and $a_{g}$ is the gender-specific effect of age on income. Income is then modelled using an ordered-logistic regression with j−1 cutpoints $\kappa_{j}$:


$$\begin{array}{c} {\hat{w}}_{i}\sim f\left(j|\theta ,\kappa \right)=\left\{ \begin{array}{c} @c\text{1}-{logit}^{-\text{1}}\left(\theta -\kappa \right),ifj=\text{0}\\ {logit}^{-\text{1}}\left(\theta -\kappa_{j-\text{1}}\right)-{logit}^{-\text{1}}\left(\theta -\kappa_{j}\right),if\text{0}<j<J\\ {logit}^{-\text{1}}\left(\theta -\kappa_{j-\text{1}}\right),j=J \end{array}\right. \end{array}$$

The cutpoints $\kappa_{j}$ define the thresholds between the ordered income categories, ranging from the lowest (£0–£300) to the highest (£1,112 or more).

**The outcome model:** Mental health outcomes are measured at the item level using ordered responses from the PHQ-9 and GAD-7 instruments. For each item q and gender g, the linear predictor for mental health is


$$\begin{array}{c} \eta_{i}=\alpha_{\text{2}\left(q,g\right)}+c_{\left(q,g\right)}\times age_{z\left(i\right)}+b_{\left(q,g\right)}\times \sum \left(j=\text{1}\right)\left(W_{i}-\text{1}\right)\delta_{j}+U_{\text{2}}^{T}X_{\text{2}\left(i\right)} \end{array}$$

Here, $\alpha_{\text{2}\left(q,g\right)}$ is an intercept varying over question and gender, $c_{\left(q,g\right)}$ captures the direct effect of age on mental health, and $b_{\left(q,g\right)}$ represents the effect of income on mental health. Income enters the model through a weighted cumulative function of income categories, where the weights $\delta_{j}$ follow a Dirichlet prior distribution. This specification allows the effect of income to increase monotonically across income levels without imposing equal spacing between categories. Finally, $U_{\text{2}}^{T}X_{\text{2}\left(i\right)}$ is the adjustment set containing education (no-tertiary, tertiary), employment (unemployed, employed), children (no, yes), religion (non-religious, religious), politics (median split: left-leaning, right-leaning), loneliness (does not meet criteria, meets criteria), housing (does not own house, owns house). All these variables were coded as binary in the C19PRC study or binarised by us for consistency and simplicity (all codes in our online repository). For the present study we were not interested in the effects of these confounders, so we treated them as nuisance parameters, and as such we included them in the confounder’s matrix *X* in a dot product with parameter *U* (size = [*confounder*_*1*_*,…, confounder*_*7*_]).

Mental health responses are modelled using an ordered-logistic likelihood over K−1 cutpoints, $\kappa_{k}$, corresponding to the four responses options for each PHQ-9 and GAD-7 item (“Not at all” to “Nearly every day”). All intercepts and slopes are estimated using a non-centred hierarchical parameterisation, allowing effects to vary by gender and by item. The subscript g indexes gender (female, male), q indexes questionnaire items (9 for PHQ-9 and 7 for GAD-7), and i indexes participants over both Wave-1 and Wave-6.

We used PyMC v5 [42] to build and sample models with the No-U Turn Sampling (NUTS) Hamiltonian Monte Carlo (HMC) method. All models sampled well, showing very good convergence, with all parameters showing effective sample sizes > 800 and $\hat{R}s\leq \text{1}.\text{01}$. Fit measures for models applied to PHQ-9 scores show mean absolute errors (MAEs) of 0.73 and 0.76 for Wave-1 and Wave-6 respectively, indicating that predicted scores are at most ±0.73 and ±0.76 scores around the true value. Concordance scores suggest that 81% of predictions are within one score (category) of the true value for Wave-1 and Wave-6 respectively. Fit measures from models applied to GAD-7 scores indicate MAEs of 0.89 and 0.79 and concordance of 77% and 81% for Wave-1 and Wave-6 respectively.

To assess posterior distributions, we used average marginal effects (AMEs) for continuous values (i.e., age), average discrete effects (ADEs) for ordinal variables (i.e., income), and average partial effects (APEs) to express the effects of income across age (for details on the calculations of these measures see: [43,44]. We defined the income effect on depression or anxiety as the probability difference of getting a certain score on any PHQ-9 or GAD-7 item between two income groups averaged across age. The age effect on depression or anxiety is presented with the probability difference of getting a certain score on any PHQ-9 or GAD-7 item between the lowest and the highest age in the sample, whereas the age effect on income is defined as the unit percentage change of income induced by 1 year of age increase. We also reported the posterior predictive probabilities of classification into each depression/anxiety severity level (i.e., clinical thresholds: minimal, mild, moderate, severe) based on the total PHQ-9 and GAD-7 sum scores.

### Significance statement

This research provides novel, longitudinal evidence on how the COVID-19 pandemic differentially shaped the critical relationship between household income and mental health across genders. Using robust Bayesian mediation models, we show that while men exhibited a stronger overall income-mental health link, this association intensified more sharply for women as the crisis progressed. These findings reveal the dynamic nature of socioeconomic disparities in psychological distress during a public health emergency, offering crucial evidence for designing gender-sensitive economic and mental health interventions in future crises.

## Supporting information

S1 Appendix Table A. PHQ-9 Analysis Effects Summary by Question (Wave-1). Table B. PHQ-9 Analysis Effects Summary by Question (Wave-6). Table C. GAD-7 Analysis Effects Summary by Question (Wave-1). Table D. GAD-7 Analysis Effects Summary by Question (Wave-6).
(DOCX)

S2 AppendixFig A. Prior predictive checks for Wave-1 and Wave-6 (Depression, PHQ-9, models).Both distributions are right-skewed, as intended from prior parameters. Fig B. Prior predictive checks for Wave-1 and Wave-6 (Anxiety, GAD-7, models). Both distributions are right-skewed, as intended from prior parameters. Fig C. Wave-1 female PHQ-9 answering probabilities posterior distribution means. (A) Probabilities of answering “Not at all” (score = 0). (B) Probabilities of answering “Several days” (score = 1). (C) Probabilities of answering “More than half the days” (score = 2). (D) Probabilities of answering “Nearly every day” (score = 3). Inc1, Inc2, Inc3, Inc4 Inc5 correspond to respective income levels: £0 - £300, £301 - £490, £491 - £740, £741 - £1,111, £1,112 + . Fig D. Wave-1 male PHQ-9 answering probabilities posterior distribution means. (A) Probabilities of answering “Not at all” (score = 0). (B) Probabilities of answering “Several days” (score = 1). (C) Probabilities of answering “More than half the days” (score = 2). (D) Probabilities of answering “Nearly every day” (score = 3). Inc1, Inc2, Inc3, Inc4 Inc5 correspond to respective income levels: £0 - £300, £301 - £490, £491 - £740, £741 - £1,111, £1,112 + . Fig E. Wave-6 female PHQ-9 answering probabilities posterior distribution means. (A) Probabilities of answering “Not at all” (score = 0). (B) Probabilities of answering “Several days” (score = 1). (C) Probabilities of answering “More than half the days” (score = 2). (D) Probabilities of answering “Nearly every day” (score = 3). Inc1, Inc2, Inc3, Inc4 Inc5 correspond to respective income levels: £0 - £300, £301 - £490, £491 - £740, £741 - £1,111, £1,112 + . Fig F. Wave-6 male PHQ-9 answering probabilities posterior distribution means. (A) Probabilities of answering “Not at all” (score = 0). (B) Probabilities of answering “Several days” (score = 1). (C) Probabilities of answering “More than half the days” (score = 2). (D) Probabilities of answering “Nearly every day” (score = 3). Inc1, Inc2, Inc3, Inc4 Inc5 correspond to respective income levels: £0 - £300, £301 - £490, £491 - £740, £741 - £1,111, £1,112 + . Fig G. Wave-1 female GAD-7 answering probabilities posterior distribution means. (A) Probabilities of answering “Not at all” (score = 0). (B) Probabilities of answering “Several days” (score = 1). (C) Probabilities of answering “More than half the days” (score = 2). (D) Probabilities of answering “Nearly every day” (score = 3). Inc1, Inc2, Inc3, Inc4 Inc5 correspond to respective income levels: £0 - £300, £301 - £490, £491 - £740, £741 - £1,111, £1,112 + . Fig H. Wave-1 male GAD-7 answering probabilities posterior distribution means. (A) Probabilities of answering “Not at all” (score = 0). (B) Probabilities of answering “Several days” (score = 1). (C) Probabilities of answering “More than half the days” (score = 2). (D) Probabilities of answering “Nearly every day” (score = 3). Inc1, Inc2, Inc3, Inc4 Inc5 correspond to respective income levels: £0 - £300, £301 - £490, £491 - £740, £741 - £1,111, £1,112 + . Fig I. Wave-6 female GAD-7 answering probabilities posterior distribution means. (A) Probabilities of answering “Not at all” (score = 0). (B) Probabilities of answering “Several days” (score = 1). (C) Probabilities of answering “More than half the days” (score = 2). (D) Probabilities of answering “Nearly every day” (score = 3). Inc1, Inc2, Inc3, Inc4 Inc5 correspond to respective income levels: £0 - £300, £301 - £490, £491 - £740, £741 - £1,111, £1,112 + . Fig J. Wave-6 male GAD-7 answering probabilities posterior distribution means. (A) Probabilities of answering “Not at all” (score = 0). (B) Probabilities of answering “Several days” (score = 1). (C) Probabilities of answering “More than half the days” (score = 2). (D) Probabilities of answering “Nearly every day” (score = 3). Inc1, Inc2, Inc3, Inc4 Inc5 correspond to respective income levels: £0 - £300, £301 - £490, £491 - £740, £741 - £1,111, £1,112 + . Fig K. Posterior predictive checks from sampled mediation model for Wave-1. Plots show posterior predictive distributions for w, likelihood of age effect on income estimation; and for y, likelihood of the effects of age and income on PHQ-9 scores estimation. Fig L. Posterior predictive checks from sampled mediation model for Wave-6. Plots show posterior predictive distributions for w, likelihood of age effect on income estimation; and for y, likelihood of the effects of age and income on PHQ-9 scores estimation. Fig M. Posterior predictive checks from sampled mediation model for Wave-1. Plots show posterior predictive distributions for $\hat{w}$, likelihood of age effect on income estimation; and for $\hat{y}$, likelihood of the effects of age and income on GAD-7 scores estimation. Fig N. Posterior predictive checks from sampled mediation model for Wave-6. Plots show posterior predictive distributions for $\hat{w}$, likelihood of age effect on income estimation; and for $\hat{y}$, likelihood of the effects of age and income on GAD-7 scores estimation.(DOCX)
