# Supplementary material for: A Bayesian approach for exploring the effects of household income on self-reported mental health measures during the COVID-19 pandemic
Source: PLOS Ment Health. 2026 Aug 25;3(8):e0000691. doi: 10.1371/journal.pmen.0000691 (PMC13505949; doi:10.1371/journal.pmen.0000691)
Supplement: Supplementary file 1 [file pmen.0000691.s001.docx]

**A Bayesian approach for exploring the effects of household income on self-reported mental health measures during the COVID-19 pandemic**

Simon Busch-Moreno^1, 2†^, Xiao Fu^3*†^, Etienne B. Roesch^4,5^

^1 Center for the Well-Being and Development of Adolescents and Children in the Digital Age (BAND). Pontificia Universidad Católica de Chile, Santiago de Chile, Chile.^

^2 Institute of Science and Innovation in Medicine (ICIM). Universidad del Desarrollo, Santiago de Chile, Chile.^

^3 Department of Computer Science and Engineering, College of Engineering, Southern University of Science and Technology, Shenzhen, China. fux3@sustech.edu.cn^

^4 School of Psychology and Clinical Language Sciences, University of Reading, Reading, U.K.^

^5 Centre for Integrative Neuroscience and Neurodynamics (CINN), University of Reading, Reading, U.K.^

^† These authors contributed equally to this work and should be considered co-first authors.^

**S1 Appendix**

The present annex displays additional tables containing summaries of effects on PHQ-9 questions (Table A and B) and on GAD-7 questions (Table C and D) in the two waves (Wave-1 and Wave-6). Effects correspond to average marginal effects for continuous variables (i.e., age) and average discrete effects for discrete variables (i.e., income) were computed from the whole posterior distribution. Tables display Mean (posterior mean), SD (posterior standard deviation), HDI 5% and HDI 95% (90% highest density interval from the posterior distribution), Effect (Mediator = Income, Direct = Age, Indirect = Age to mental health measures through Income, Total = Direct+Indirect), Gender (coded as Female and Male in original study), and Question (every PHQ-9 or GAD-7 questionnaire item).

| **Table A. PHQ-9 Analysis Effects Summary by Question (Wave-1)** | | | | | | |
| --- | --- | --- | --- | --- | --- | --- |
| **Mean** | **SD** | **HDI 5%** | **HDI 95%** | **Effect** | **Sex** | **Question** |
| -0.044 | 0.056 | -0.188 | 0.012 | Mediator | Female | Q1 |
| 0.042 | 0.06 | 0.049 | 0.192 | Mediator | Male | Q1 |
| -0.078 | 0.089 | 0.056 | 0.34 | Direct | Female | Q1 |
| 0.08 | 0.093 | 0.08 | 0.427 | Direct | Male | Q1 |
| 0.00019 | 0.00046 | -0.00031 | 0.00132 | Indirect | Female | Q1 |
| -0.00035 | 0.00075 | -0.00271 | 0.00036 | Indirect | Male | Q1 |
| -0.078 | 0.089 | 0.057 | 0.339 | Total | Female | Q1 |
| 0.08 | 0.093 | 0.08 | 0.426 | Total | Male | Q1 |
| -0.067 | 0.06 | 0.053 | 0.241 | Mediator | Female | Q2 |
| 0.067 | 0.059 | -0.134 | 0.036 | Mediator | Male | Q2 |
| 0.164 | 0.088 | 0.213 | 0.567 | Direct | Female | Q2 |
| 0.27 | 0.1 | 0.016 | 0.294 | Direct | Male | Q2 |
| 0.0003 | 0.0005 | -0.00327 | 0.0004 | Indirect | Female | Q2 |
| -0.00061 | 0.00085 | -0.00039 | 0.0009 | Indirect | Male | Q2 |
| 0.164 | 0.088 | 0.213 | 0.566 | Total | Female | Q2 |
| 0.269 | 0.1 | 0.018 | 0.295 | Total | Male | Q2 |
| -0.079 | 0.055 | -0.13 | 0.054 | Mediator | Female | Q3 |
| 0.049 | 0.045 | 0.032 | 0.198 | Mediator | Male | Q3 |
| 0.278 | 0.096 | -0.208 | 0.079 | Direct | Female | Q3 |
| 0.15 | 0.088 | 0.083 | 0.417 | Direct | Male | Q3 |
| 0.00034 | 0.00049 | -0.00042 | 0.00084 | Indirect | Female | Q3 |
| -0.00044 | 0.00066 | -0.00262 | 0.00042 | Indirect | Male | Q3 |
| 0.279 | 0.097 | -0.21 | 0.077 | Total | Female | Q3 |
| 0.149 | 0.088 | 0.084 | 0.417 | Total | Male | Q3 |
| -0.053 | 0.054 | -0.163 | 0.032 | Mediator | Female | Q4 |
| 0.12 | 0.051 | 0.005 | 0.098 | Mediator | Male | Q4 |
| 0.154 | 0.085 | 0.006 | 0.3 | Direct | Female | Q4 |
| 0.258 | 0.105 | 0.02 | 0.322 | Direct | Male | Q4 |
| 0.00022 | 0.00045 | -0.00038 | 0.00106 | Indirect | Female | Q4 |
| -0.00105 | 0.00108 | -0.00122 | 0.00027 | Indirect | Male | Q4 |
| 0.154 | 0.085 | 0.007 | 0.301 | Total | Female | Q4 |
| 0.257 | 0.105 | 0.021 | 0.323 | Total | Male | Q4 |
| -0.01 | 0.028 | -0.03 | 0.157 | Mediator | Female | Q5 |
| 0.051 | 0.03 | -0.028 | 0.047 | Mediator | Male | Q5 |
| 0.06 | 0.057 | 0.113 | 0.433 | Direct | Female | Q5 |
| 0.182 | 0.101 | 0.018 | 0.237 | Direct | Male | Q5 |
| 5.00E-05 | 0.0002 | -0.00203 | 0.00037 | Indirect | Female | Q5 |
| -0.00045 | 0.00055 | -0.00026 | 0.00018 | Indirect | Male | Q5 |
| 0.06 | 0.057 | 0.111 | 0.434 | Total | Female | Q5 |
| 0.181 | 0.101 | 0.017 | 0.238 | Total | Male | Q5 |
| 0.004 | 0.024 | -0.17 | 0.008 | Mediator | Female | Q6 |
| 0.098 | 0.041 | 0.037 | 0.162 | Mediator | Male | Q6 |
| 0.128 | 0.08 | 0.141 | 0.458 | Direct | Female | Q6 |
| 0.168 | 0.094 | 0.032 | 0.315 | Direct | Male | Q6 |
| -2.00E-05 | 0.00014 | -0.00035 | 0.00116 | Indirect | Female | Q6 |
| -0.00086 | 0.00087 | -0.00237 | 0.00031 | Indirect | Male | Q6 |
| 0.128 | 0.08 | 0.141 | 0.458 | Total | Female | Q6 |
| 0.167 | 0.094 | 0.031 | 0.313 | Total | Male | Q6 |
| -0.082 | 0.062 | -0.03 | 0.148 | Mediator | Female | Q7 |
| 0.137 | 0.057 | -0.077 | 0.053 | Mediator | Male | Q7 |
| 0.204 | 0.088 | 0.159 | 0.487 | Direct | Female | Q7 |
| 0.39 | 0.108 | 0.121 | 0.449 | Direct | Male | Q7 |
| 0.00035 | 0.00057 | -0.0018 | 0.00047 | Indirect | Female | Q7 |
| -0.00121 | 0.00124 | -0.0003 | 0.00042 | Indirect | Male | Q7 |
| 0.204 | 0.088 | 0.157 | 0.485 | Total | Female | Q7 |
| 0.388 | 0.108 | 0.121 | 0.449 | Total | Male | Q7 |
| -0.013 | 0.041 | -0.051 | 0.147 | Mediator | Female | Q8 |
| 0.117 | 0.045 | -0.049 | 0.032 | Mediator | Male | Q8 |
| 0.288 | 0.102 | -0.079 | 0.221 | Direct | Female | Q8 |
| 0.24 | 0.11 | -0.024 | 0.145 | Direct | Male | Q8 |
| 6.00E-05 | 0.00029 | -0.00148 | 0.00081 | Indirect | Female | Q8 |
| -0.00103 | 0.00103 | -0.00022 | 0.00031 | Indirect | Male | Q8 |
| 0.288 | 0.102 | -0.079 | 0.221 | Total | Female | Q8 |
| 0.239 | 0.11 | -0.024 | 0.145 | Total | Male | Q8 |
| -0.053 | 0.056 | -0.149 | 0.033 | Mediator | Female | Q9 |
| 0.06 | 0.054 | -0.022 | 0.126 | Mediator | Male | Q9 |
| 0.207 | 0.087 | 0.074 | 0.354 | Direct | Female | Q9 |
| 0.327 | 0.102 | 0.003 | 0.278 | Direct | Male | Q9 |
| 0.00024 | 0.00044 | -0.00027 | 0.00102 | Indirect | Female | Q9 |
| -0.00051 | 0.00078 | -0.0014 | 0.00041 | Indirect | Male | Q9 |
| 0.207 | 0.087 | 0.076 | 0.355 | Total | Female | Q9 |
| 0.326 | 0.102 | 0.002 | 0.277 | Total | Male | Q9 |

| **Table B. PHQ-9 Analysis Effects Summary by Question (Wave-6)** | | | | | | |
| --- | --- | --- | --- | --- | --- | --- |
| **Mean** | **SD** | **HDI 5%** | **HDI 95%** | **Effect** | **Sex** | **Question** |
| 0.12 | 0.049 | -0.014 | 0.165 | Mediator | Female | Q1 |
| 0.087 | 0.054 | 0.013 | 0.156 | Mediator | Male | Q1 |
| 0.094 | 0.077 | 0.015 | 0.336 | Direct | Female | Q1 |
| 0.113 | 0.09 | 0.1 | 0.414 | Direct | Male | Q1 |
| -0.00096 | 0.00082 | -0.00355 | 0.00069 | Indirect | Female | Q1 |
| -0.00176 | 0.00146 | -0.00152 | 0.00022 | Indirect | Male | Q1 |
| 0.093 | 0.077 | -0.003 | 0.32 | Total | Female | Q1 |
| 0.111 | 0.09 | 0.099 | 0.413 | Total | Male | Q1 |
| 0.12 | 0.052 | 0.005 | 0.177 | Mediator | Female | Q2 |
| 0.079 | 0.058 | 0.028 | 0.183 | Mediator | Male | Q2 |
| 0.163 | 0.087 | -0.021 | 0.266 | Direct | Female | Q2 |
| 0.128 | 0.095 | -0.003 | 0.223 | Direct | Male | Q2 |
| -0.00095 | 0.00082 | -0.00417 | 0.00014 | Indirect | Female | Q2 |
| -0.00159 | 0.00144 | -0.00194 | 7.00E-05 | Indirect | Male | Q2 |
| 0.162 | 0.087 | -0.023 | 0.264 | Total | Female | Q2 |
| 0.127 | 0.095 | -0.003 | 0.223 | Total | Male | Q2 |
| 0.07 | 0.046 | 0.036 | 0.203 | Mediator | Female | Q3 |
| 0.061 | 0.048 | -0.002 | 0.158 | Mediator | Male | Q3 |
| 0.109 | 0.078 | -0.243 | 0.03 | Direct | Female | Q3 |
| 0.196 | 0.1 | 0.106 | 0.44 | Direct | Male | Q3 |
| -0.00055 | 0.00056 | -0.00222 | 0.00022 | Indirect | Female | Q3 |
| -0.00122 | 0.0012 | -0.004 | 0.0001 | Indirect | Male | Q3 |
| 0.108 | 0.078 | -0.244 | 0.029 | Total | Female | Q3 |
| 0.194 | 0.1 | 0.104 | 0.438 | Total | Male | Q3 |
| 0.112 | 0.046 | 0.039 | 0.203 | Mediator | Female | Q4 |
| 0.084 | 0.05 | -0.034 | 0.082 | Mediator | Male | Q4 |
| 0.102 | 0.072 | 0.015 | 0.298 | Direct | Female | Q4 |
| 0.273 | 0.101 | 0.024 | 0.292 | Direct | Male | Q4 |
| -0.0009 | 0.00078 | -0.00213 | 0.00017 | Indirect | Female | Q4 |
| -0.0017 | 0.00138 | -0.00187 | 0.00069 | Indirect | Male | Q4 |
| 0.101 | 0.072 | 0.015 | 0.298 | Total | Female | Q4 |
| 0.272 | 0.101 | 0.024 | 0.292 | Total | Male | Q4 |
| 0.03 | 0.028 | -0.014 | 0.177 | Mediator | Female | Q5 |
| 0.027 | 0.035 | 0.008 | 0.087 | Mediator | Male | Q5 |
| 0.071 | 0.057 | -0.017 | 0.291 | Direct | Female | Q5 |
| 0.153 | 0.088 | 0.018 | 0.223 | Direct | Male | Q5 |
| -0.00025 | 0.00033 | -0.00361 | 0.00062 | Indirect | Female | Q5 |
| -0.00056 | 0.00084 | -0.00087 | 8.00E-05 | Indirect | Male | Q5 |
| 0.071 | 0.057 | -0.019 | 0.289 | Total | Female | Q5 |
| 0.153 | 0.088 | 0.018 | 0.223 | Total | Male | Q5 |
| 0.046 | 0.027 | -0.003 | 0.149 | Mediator | Female | Q6 |
| 0.017 | 0.035 | -0.032 | 0.076 | Mediator | Male | Q6 |
| 0.12 | 0.07 | -0.005 | 0.242 | Direct | Female | Q6 |
| 0.196 | 0.102 | 0.041 | 0.35 | Direct | Male | Q6 |
| -0.00036 | 0.00035 | -0.00151 | 0.00017 | Indirect | Female | Q6 |
| -0.00032 | 0.0008 | -0.00159 | 0.00077 | Indirect | Male | Q6 |
| 0.12 | 0.07 | -0.005 | 0.241 | Total | Female | Q6 |
| 0.196 | 0.102 | 0.041 | 0.35 | Total | Male | Q6 |
| 0.085 | 0.045 | -0.046 | 0.14 | Mediator | Female | Q7 |
| 0.107 | 0.051 | -0.013 | 0.074 | Mediator | Male | Q7 |
| 0.259 | 0.097 | -0.1 | 0.238 | Direct | Female | Q7 |
| 0.231 | 0.102 | -0.003 | 0.161 | Direct | Male | Q7 |
| -0.00068 | 0.00061 | -0.00311 | 0.00106 | Indirect | Female | Q7 |
| -0.00218 | 0.00158 | -0.00075 | 0.00012 | Indirect | Male | Q7 |
| 0.258 | 0.097 | -0.102 | 0.235 | Total | Female | Q7 |
| 0.229 | 0.102 | -0.002 | 0.162 | Total | Male | Q7 |
| 0.13 | 0.052 | 0.035 | 0.199 | Mediator | Female | Q8 |
| 0.053 | 0.058 | 0.022 | 0.19 | Mediator | Male | Q8 |
| -0.108 | 0.085 | -0.03 | 0.221 | Direct | Female | Q8 |
| 0.064 | 0.103 | 0.074 | 0.393 | Direct | Male | Q8 |
| -0.00103 | 0.00083 | -0.00212 | 0.0002 | Indirect | Female | Q8 |
| -0.00107 | 0.00137 | -0.00457 | -1.00E-05 | Indirect | Male | Q8 |
| -0.109 | 0.085 | -0.026 | 0.225 | Total | Female | Q8 |
| 0.063 | 0.103 | 0.075 | 0.393 | Total | Male | Q8 |
| 0.172 | 0.052 | 0.082 | 0.253 | Mediator | Female | Q9 |
| 0.071 | 0.055 | -0.026 | 0.131 | Mediator | Male | Q9 |
| 0.071 | 0.075 | -0.049 | 0.191 | Direct | Female | Q9 |
| 0.16 | 0.098 | 0.036 | 0.339 | Direct | Male | Q9 |
| -0.0014 | 0.00114 | -0.00298 | 0.0003 | Indirect | Female | Q9 |
| -0.00145 | 0.00137 | -0.0031 | 0.00047 | Indirect | Male | Q9 |
| 0.07 | 0.075 | -0.048 | 0.192 | Total | Female | Q9 |
| 0.159 | 0.099 | 0.033 | 0.337 | Total | Male | Q9 |

| **Table C. GAD-7 Analysis Effects Summary by Question (Wave-1)** | | | | | | |
| --- | --- | --- | --- | --- | --- | --- |
| **Mean** | **SD** | **HDI 5%** | **HDI 95%** | **Effect** | **Sex** | **Question** |
| -0.049 | 0.056 | 0.062 | 0.257 | Mediator | Female | Q1 |
| 0.178 | 0.056 | -0.143 | 0.006 | Mediator | Male | Q1 |
| 0.257 | 0.087 | 0.176 | 0.503 | Direct | Female | Q1 |
| 0.307 | 0.105 | 0.023 | 0.283 | Direct | Male | Q1 |
| 0.00022 | 0.00047 | -0.00379 | 0.0007 | Indirect | Female | Q1 |
| -0.00149 | 0.00157 | -0.00033 | 0.00104 | Indirect | Male | Q1 |
| 0.257 | 0.087 | 0.175 | 0.503 | Total | Female | Q1 |
| 0.306 | 0.105 | 0.023 | 0.283 | Total | Male | Q1 |
| -0.059 | 0.057 | -0.144 | 0.037 | Mediator | Female | Q2 |
| 0.189 | 0.057 | 0.054 | 0.22 | Mediator | Male | Q2 |
| 0.269 | 0.085 | 0.118 | 0.4 | Direct | Female | Q2 |
| 0.394 | 0.106 | 0.139 | 0.512 | Direct | Male | Q2 |
| 0.00026 | 0.0005 | -0.00033 | 0.00102 | Indirect | Female | Q2 |
| -0.00155 | 0.00159 | -0.00299 | 0.00071 | Indirect | Male | Q2 |
| 0.269 | 0.085 | 0.118 | 0.4 | Total | Female | Q2 |
| 0.392 | 0.107 | 0.139 | 0.51 | Total | Male | Q2 |
| -0.062 | 0.047 | -0.149 | 0.036 | Mediator | Female | Q3 |
| 0.134 | 0.052 | 0.039 | 0.219 | Mediator | Male | Q3 |
| 0.134 | 0.081 | 0.128 | 0.407 | Direct | Female | Q3 |
| 0.337 | 0.116 | 0.225 | 0.57 | Direct | Male | Q3 |
| 0.00028 | 0.0005 | -0.00051 | 0.001 | Indirect | Female | Q3 |
| -0.00113 | 0.0012 | -0.00291 | 0.00052 | Indirect | Male | Q3 |
| 0.134 | 0.081 | 0.128 | 0.407 | Total | Female | Q3 |
| 0.336 | 0.116 | 0.223 | 0.569 | Total | Male | Q3 |
| -0.097 | 0.054 | -0.142 | 0.028 | Mediator | Female | Q4 |
| 0.113 | 0.055 | 0.029 | 0.203 | Mediator | Male | Q4 |
| 0.041 | 0.093 | 0.053 | 0.33 | Direct | Female | Q4 |
| 0.234 | 0.096 | 0.097 | 0.415 | Direct | Male | Q4 |
| 0.00045 | 0.00073 | -0.00042 | 0.00099 | Indirect | Female | Q4 |
| -0.00093 | 0.00109 | -0.00252 | 0.00068 | Indirect | Male | Q4 |
| 0.042 | 0.093 | 0.053 | 0.331 | Total | Female | Q4 |
| 0.233 | 0.096 | 0.095 | 0.413 | Total | Male | Q4 |
| -0.052 | 0.056 | 0.096 | 0.277 | Mediator | Female | Q5 |
| 0.122 | 0.055 | -0.183 | -0.004 | Mediator | Male | Q5 |
| 0.293 | 0.087 | 0.233 | 0.584 | Direct | Female | Q5 |
| 0.404 | 0.104 | -0.112 | 0.194 | Direct | Male | Q5 |
| 0.00023 | 0.00047 | -0.00411 | 0.00092 | Indirect | Female | Q5 |
| -0.00103 | 0.00118 | -0.00068 | 0.0015 | Indirect | Male | Q5 |
| 0.293 | 0.087 | 0.232 | 0.581 | Total | Female | Q5 |
| 0.403 | 0.104 | -0.111 | 0.194 | Total | Male | Q5 |
| -0.055 | 0.053 | 0.081 | 0.265 | Mediator | Female | Q6 |
| 0.145 | 0.054 | -0.146 | 0.034 | Mediator | Male | Q6 |
| 0.19 | 0.086 | 0.144 | 0.483 | Direct | Female | Q6 |
| 0.323 | 0.103 | 0.169 | 0.443 | Direct | Male | Q6 |
| 0.00025 | 0.00049 | -0.00377 | 0.0012 | Indirect | Female | Q6 |
| -0.00122 | 0.00134 | -0.00045 | 0.00095 | Indirect | Male | Q6 |
| 0.19 | 0.086 | 0.146 | 0.486 | Total | Female | Q6 |
| 0.322 | 0.103 | 0.17 | 0.443 | Total | Male | Q6 |
| -0.094 | 0.056 | -0.185 | -0.002 | Mediator | Female | Q7 |
| 0.165 | 0.059 | 0.055 | 0.234 | Mediator | Male | Q7 |
| 0.332 | 0.08 | 0.201 | 0.46 | Direct | Female | Q7 |
| 0.34 | 0.1 | 0.148 | 0.48 | Direct | Male | Q7 |
| 0.00043 | 0.00065 | -0.00052 | 0.00144 | Indirect | Female | Q7 |
| -0.00137 | 0.0015 | -0.00319 | 0.00073 | Indirect | Male | Q7 |
| 0.333 | 0.08 | 0.202 | 0.46 | Total | Female | Q7 |
| 0.339 | 0.1 | 0.146 | 0.48 | Total | Male | Q7 |

| **Table D. GAD-7 Analysis Effects Summary by Question (Wave-6)** | | | | | | |
| --- | --- | --- | --- | --- | --- | --- |
| **Mean** | **SD** | **HDI 5%** | **HDI 95%** | **Effect** | **Sex** | **Question** |
| 0.092 | 0.045 | 0.046 | 0.231 | Mediator | Female | Q1 |
| 0.127 | 0.051 | -0.002 | 0.119 | Mediator | Male | Q1 |
| 0.158 | 0.081 | 0.071 | 0.396 | Direct | Female | Q1 |
| 0.2 | 0.098 | -0.022 | 0.171 | Direct | Male | Q1 |
| -0.00076 | 0.00074 | -0.00546 | -0.00019 | Indirect | Female | Q1 |
| -0.0025 | 0.00161 | -0.00124 | 0.00016 | Indirect | Male | Q1 |
| 0.158 | 0.081 | 0.067 | 0.393 | Total | Female | Q1 |
| 0.197 | 0.098 | -0.023 | 0.17 | Total | Male | Q1 |
| 0.098 | 0.053 | 0.01 | 0.159 | Mediator | Female | Q2 |
| 0.145 | 0.052 | -0.066 | 0.079 | Mediator | Male | Q2 |
| 0.17 | 0.087 | 0.024 | 0.285 | Direct | Female | Q2 |
| 0.233 | 0.098 | 0.061 | 0.412 | Direct | Male | Q2 |
| -0.00081 | 0.00081 | -0.00192 | 0.00019 | Indirect | Female | Q2 |
| -0.00291 | 0.00184 | -0.002 | 0.00109 | Indirect | Male | Q2 |
| 0.169 | 0.087 | 0.023 | 0.284 | Total | Female | Q2 |
| 0.23 | 0.098 | 0.062 | 0.412 | Total | Male | Q2 |
| 0.061 | 0.038 | 0.006 | 0.18 | Mediator | Female | Q3 |
| 0.013 | 0.045 | 0.012 | 0.179 | Mediator | Male | Q3 |
| 0.07 | 0.062 | 0.045 | 0.334 | Direct | Female | Q3 |
| 0.248 | 0.109 | -0.028 | 0.311 | Direct | Male | Q3 |
| -0.00048 | 0.00048 | -0.00203 | 0.00018 | Indirect | Female | Q3 |
| -0.00025 | 0.00098 | -0.00421 | 5.00E-05 | Indirect | Male | Q3 |
| 0.07 | 0.063 | 0.043 | 0.332 | Total | Female | Q3 |
| 0.248 | 0.11 | -0.028 | 0.31 | Total | Male | Q3 |
| 0.059 | 0.049 | 0.068 | 0.23 | Mediator | Female | Q4 |
| 0.093 | 0.051 | 0.043 | 0.185 | Mediator | Male | Q4 |
| 0.201 | 0.085 | 0.086 | 0.408 | Direct | Female | Q4 |
| 0.138 | 0.102 | -0.019 | 0.203 | Direct | Male | Q4 |
| -0.00049 | 0.00059 | -0.0057 | -0.00031 | Indirect | Female | Q4 |
| -0.00186 | 0.00144 | -0.00204 | 0.00028 | Indirect | Male | Q4 |
| 0.201 | 0.085 | 0.085 | 0.405 | Total | Female | Q4 |
| 0.136 | 0.102 | -0.026 | 0.197 | Total | Male | Q4 |
| 0.112 | 0.045 | 0.032 | 0.191 | Mediator | Female | Q5 |
| 0.119 | 0.047 | 0.038 | 0.19 | Mediator | Male | Q5 |
| 0.089 | 0.069 | -0.037 | 0.233 | Direct | Female | Q5 |
| 0.16 | 0.093 | 0.024 | 0.313 | Direct | Male | Q5 |
| -0.00091 | 0.00083 | -0.00207 | 0.00025 | Indirect | Female | Q5 |
| -0.00236 | 0.00154 | -0.00468 | -0.00024 | Indirect | Male | Q5 |
| 0.088 | 0.069 | -0.016 | 0.254 | Total | Female | Q5 |
| 0.157 | 0.092 | 0.02 | 0.308 | Total | Male | Q5 |
| 0.106 | 0.05 | 0.051 | 0.217 | Mediator | Female | Q6 |
| 0.119 | 0.054 | -0.013 | 0.149 | Mediator | Male | Q6 |
| 0.102 | 0.083 | 0.041 | 0.359 | Direct | Female | Q6 |
| 0.213 | 0.097 | 0.068 | 0.344 | Direct | Male | Q6 |
| -0.00087 | 0.00084 | -0.005 | -0.00034 | Indirect | Female | Q6 |
| -0.00239 | 0.00172 | -0.00141 | 0.00032 | Indirect | Male | Q6 |
| 0.101 | 0.083 | 0.04 | 0.359 | Total | Female | Q6 |
| 0.211 | 0.097 | 0.065 | 0.341 | Total | Male | Q6 |
| 0.093 | 0.05 | 0.01 | 0.171 | Mediator | Female | Q7 |
| 0.143 | 0.056 | 0.037 | 0.21 | Mediator | Male | Q7 |
| 0.185 | 0.087 | 0.05 | 0.335 | Direct | Female | Q7 |
| 0.225 | 0.101 | 0.064 | 0.381 | Direct | Male | Q7 |
| -0.00076 | 0.00073 | -0.00186 | 0.00021 | Indirect | Female | Q7 |
| -0.00284 | 0.00187 | -0.00508 | 7.00E-05 | Indirect | Male | Q7 |
| 0.185 | 0.087 | 0.05 | 0.334 | Total | Female | Q7 |
| 0.222 | 0.101 | 0.063 | 0.381 | Total | Male | Q7 |
