# Supplementary material for: A Bayesian approach for exploring the effects of household income on self-reported mental health measures during the COVID-19 pandemic
Source: PLOS Ment Health. 2026 Aug 25;3(8):e0000691. doi: 10.1371/journal.pmen.0000691 (PMC13505949; doi:10.1371/journal.pmen.0000691)
Supplement: Supplementary file 2 [file pmen.0000691.s002.docx]

**A Bayesian approach for exploring the effects of household income on self-reported mental health measures during the COVID-19 pandemic**

Simon Busch-Moreno^1, 2†^, Xiao Fu^3*†^, Etienne B. Roesch^4,5^

^1 Center for the Well-Being and Development of Adolescents and Children in the Digital Age (BAND). Pontificia Universidad Católica de Chile, Santiago de Chile, Chile.^

^2 Institute of Science and Innovation in Medicine (ICIM). Universidad del Desarrollo, Santiago de Chile, Chile.^

^3 Department of Computer Science and Engineering, College of Engineering, Southern University of Science and Technology, Shenzhen, China. fux3@sustech.edu.cn^

^4 School of Psychology and Clinical Language Sciences, University of Reading, Reading, U.K.^

^5 Centre for Integrative Neuroscience and Neurodynamics (CINN), University of Reading, Reading, U.K.^

^† These authors contributed equally to this work and should be considered co-first authors.^

**S2 Appendix**

The present annex displays summaries of our Bayesian workflow approach via figures showing: 1. Prior predictive checks from all models (Fig A and B). They indicate good prior coverage and sensible prior distributions regarding income and mental health score distributions as often observed in the literature.

2. Posterior assessment plots displaying posterior distribution means of answering probabilities to each PHQ-9 question with a given score (Fig C-F), and figures containing plots displaying posterior distribution means of answering probabilities to each GAD-7 question with a given score (Fig G-J).
3. Posterior predictive distributions plots for assessing model fit (Fig K-N).

Fig A. Prior predictive checks for Wave-1 and Wave-6 (Depression, PHQ-9, models). Both distributions are right-skewed, as intended from prior parameters.

Fig B. Prior predictive checks for Wave-1 and Wave-6 (Anxiety, GAD-7, models). Both distributions are right-skewed, as intended from prior parameters.

Fig C. Wave-1 female PHQ-9 answering probabilities posterior distribution means. (A) Probabilities of answering “Not at all” (score = 0). (B) Probabilities of answering “Several days” (score = 1). (C) Probabilities of answering “More than half the days” (score = 2). (D) Probabilities of answering “Nearly every day” (score = 3). Inc1, Inc2, Inc3, Inc4 Inc5 correspond to respective income levels: £0 - £300, £301 - £490, £491 - £740, £741 - £1,111, £1,112+.

Fig D. Wave-1 male PHQ-9 answering probabilities posterior distribution means. (A) Probabilities of answering “Not at all” (score = 0). (B) Probabilities of answering “Several days” (score = 1). (C) Probabilities of answering “More than half the days” (score = 2). (D) Probabilities of answering “Nearly every day” (score = 3). Inc1, Inc2, Inc3, Inc4 Inc5 correspond to respective income levels: £0 - £300, £301 - £490, £491 - £740, £741 - £1,111, £1,112+.

Fig E. Wave-6 female PHQ-9 answering probabilities posterior distribution means. (A) Probabilities of answering “Not at all” (score = 0). (B) Probabilities of answering “Several days” (score = 1). (C) Probabilities of answering “More than half the days” (score = 2). (D) Probabilities of answering “Nearly every day” (score = 3). Inc1, Inc2, Inc3, Inc4 Inc5 correspond to respective income levels: £0 - £300, £301 - £490, £491 - £740, £741 - £1,111, £1,112+.

Fig F. Wave-6 male PHQ-9 answering probabilities posterior distribution means. (A) Probabilities of answering “Not at all” (score = 0). (B) Probabilities of answering “Several days” (score = 1). (C) Probabilities of answering “More than half the days” (score = 2). (D) Probabilities of answering “Nearly every day” (score = 3). Inc1, Inc2, Inc3, Inc4 Inc5 correspond to respective income levels: £0 - £300, £301 - £490, £491 - £740, £741 - £1,111, £1,112+.

Fig G. Wave-1 female GAD-7 answering probabilities posterior distribution means. (A) Probabilities of answering “Not at all” (score = 0). (B) Probabilities of answering “Several days” (score = 1). (C) Probabilities of answering “More than half the days” (score = 2). (D) Probabilities of answering “Nearly every day” (score = 3). Inc1, Inc2, Inc3, Inc4 Inc5 correspond to respective income levels: £0 - £300, £301 - £490, £491 - £740, £741 - £1,111, £1,112+.

Fig H. Wave-1 male GAD-7 answering probabilities posterior distribution means. (A) Probabilities of answering “Not at all” (score = 0). (B) Probabilities of answering “Several days” (score = 1). (C) Probabilities of answering “More than half the days” (score = 2). (D) Probabilities of answering “Nearly every day” (score = 3). Inc1, Inc2, Inc3, Inc4 Inc5 correspond to respective income levels: £0 - £300, £301 - £490, £491 - £740, £741 - £1,111, £1,112+.

Fig I. Wave-6 female GAD-7 answering probabilities posterior distribution means. (A) Probabilities of answering “Not at all” (score = 0). (B) Probabilities of answering “Several days” (score = 1). (C) Probabilities of answering “More than half the days” (score = 2). (D) Probabilities of answering “Nearly every day” (score = 3). Inc1, Inc2, Inc3, Inc4 Inc5 correspond to respective income levels: £0 - £300, £301 - £490, £491 - £740, £741 - £1,111, £1,112+.

Fig J. Wave-6 male GAD-7 answering probabilities posterior distribution means. (A) Probabilities of answering “Not at all” (score = 0). (B) Probabilities of answering “Several days” (score = 1). (C) Probabilities of answering “More than half the days” (score = 2). (D) Probabilities of answering “Nearly every day” (score = 3). Inc1, Inc2, Inc3, Inc4 Inc5 correspond to respective income levels: £0 - £300, £301 - £490, £491 - £740, £741 - £1,111, £1,112+.

Fig K. Posterior predictive checks from sampled mediation model for Wave-1. Plots show posterior predictive distributions for $\hat{w}$, likelihood of age effect on income estimation; and for $\hat{y}$, likelihood of the effects of age and income on PHQ-9 scores estimation.

Fig L. Posterior predictive checks from sampled mediation model for Wave-6. Plots show posterior predictive distributions for $\hat{w}$, likelihood of age effect on income estimation; and for $\hat{y}$, likelihood of the effects of age and income on PHQ-9 scores estimation.

Fig M. Posterior predictive checks from sampled mediation model for Wave-1. Plots show posterior predictive distributions for $\hat{w}$, likelihood of age effect on income estimation; and for $\hat{y}$, likelihood of the effects of age and income on GAD-7 scores estimation.

Fig N. Posterior predictive checks from sampled mediation model for Wave-6. Plots show posterior predictive distributions for $\hat{w}$, likelihood of age effect on income estimation; and for $\hat{y}$, likelihood of the effects of age and income on GAD-7 scores estimation.
