# Supplementary material for: A Bayesian approach for exploring the effects of household income on self-reported mental health measures during the COVID-19 pandemic
Source: PLOS Ment Health. 2026 Aug 25;3(8):e0000691. doi: 10.1371/journal.pmen.0000691 (PMC13505949; doi:10.1371/journal.pmen.0000691)
Supplement: Supplementary file 3 [file pmen.0000691.s004.docx]

Response to reviewers

**Reviewer #1**: This manuscript uses publicly available longitudinal data gathered from a UK sample during the COVID-19 pandemic to examine the relationship between weekly income levels and mental health in the early stage of the health crisis and 20 months later. Widely used standardized self-reported measures for depression and anxiety are used as mental health outcomes. Since income and mental health are known to vary across age and gender, the authors rely on a bayesian hierarchical framework to account for the confounding associations with these two demographic factors. More specifically, they estimate mediation models with direct effects of age on mental health and indirect effects through income levels, as well as gender-specific parameters for mental health baseline levels and the effects of income and age.

Although the analyses reported suggest a negligible mediation of age by income in all models, consistent negative effects of age and income on depression and anxiety were found. Furthermore, the analytical framework used by the authors allow them to reveal interesting age- and gender-specific patterns for income effects, that seem to have shaped participants' mental health outcomes throughout the pandemic. In particular, the depression and anxiety reducing effect of higher income was consistently greater in males than females, but showed a marked increase between the beginning of the pandemic and 20 months later which was especially strong for females.

Overall, given the manuscript's robust statistical methodology supported by publicly shared analysis codes, and provided that the authors satisfactorily address the points raised below, I would support its publication in PLOS Mental Health.

---

We really appreciate the time and effort you have taken in reviewing our manuscript. Your comments have been extremely helpful to us to improve the rigour and quality of our research. Below we have answered to your concerns one by one, explaining the changes we have made to address them and improve related issues. As this reply, you will find all other replies written in green, and all changes made referred by lines in the manuscript where they will be written in blue.

General comments

- The effects of household income levels on mental healths are said to be explored via a "Bayesian causal model", which is represented by the simple Directed Acyclic Graph (DAG) for the mediation model in figure 7. Yet, in the first sentence of the abstract the authors state that the relationship between income and mental health can be confounded by "age, gender, and other demographic factors". Therefore, do the authors consider that the coefficients they report should be interpreted causally or are there obvious unmeasured confounding variables that likely generate some bias?

Reply 1-1: We really appreciate this comment, as it has led us to carefully examine the assumptions of our model. We have conducted an additional detailed DAG exploration (dagitty code available in our draw_dag_extended.py file) to guarantee minimal sets of adjustments for effects on income and mental health. We also decided to perform **one physical adjustment and several statistical adjustments**, so we can assert the model as causal for UK’s population of white ethnicity.

**The physical adjustment:** One important demographic factor that can be a confounder is ethnicity. The representation of non-white ethnicities corresponded to only 6.4% of the total sample in our initial submission. Due to this small proportion, the data would not represent these groups well and was therefore excluded (n = 65, Line 657). After exclusion, our sample comprises 939 participants (Line 659-660).

**Statistical adjustments:** All other variables found to have meaningful causal relationships with our outcome, exposures or between them, were included as confounders adjusted in the income and mental health sub-models. These parameters are education (no-tertiary, tertiary), employment (unemployed, employed), children (no, yes), religion (non-religious, religious), political affiliation (median split: left-leaning, right-leaning), loneliness (does not meet criteria, meets criteria) and housing (does not own house, owns house) (Line 716-723). As these parameters are not of direct interest to our study, we treat them as nuisance parameters.

After re-analysis, our main conclusions have not changed. The key relationships still point in the same direction. The updated results showed that the direction of effects (AMEs and ADEs) remains the same. Although some magnitudes are now different and the uncertainty is greater, it is expected when conducting appropriate confounder adjustments. The changes have been properly reported and addressed in our results and discussion, mainly see in figures 1 to 8, the summary of the results in section 2.3 (Line 356-380) and the first two paragraphs of section 3 (Line 395-414).

We hope this addresses the reviewers’ concern in an appropriate and rigorous manner. Our thanks again for emphasising this issue, as now our analyses have greatly improved in terms of quality and interpretability, with a more straightforward causal interpretation and less biased estimates.

- The authors conclude by highlighting the value of the reported evidence to "inform targeted mental health policies and economic support programs for future emergencies". Could they explicit a bit more what type of health policies and/or resource allocation adjustments the age- and gender-specific patterns obtained may call for?

Reply 1-2: Thank you. We acknowledge this is a very important point, so we have dedicated a new section in the discussion (3.4 Implications and Future Directions, Line 597-610) to approach it. (To address this issue, we cited Adam et al., 2020, Platt et al., 2016.)

- p. 24, l. 436-438: The authors say "Accordingly, the effect of age on income was not a primary focus in the present research; instead, we examined the joint influence of age and income on mental health, as well as how the effect of income on mental health varies across age.", but this seems in contradiction with the presentation of the analytical strategy in other parts of the manuscript (e.g. lines 110-111 "our goal is to propose a methodological framework for understanding the effects of income on mental health as a potential mediator of age") and the fact that they highlight the novelty of fitting a bayesian causal mediation model (e.g. "Income effects on mental health have been widely demonstrated in the previous literature (see Thomson et al., 2022), but they have not been systematically explored, using bespoke causal models").

Reply 1-3: Many thanks for this remark. Our intention with the first sentence was to emphasise that we were not primarily interested on the direct effect of age on income. In later parts of the texts we tried to express that we explored whether income mediates the effect of age on mental health, and our main focus was how the relationship between income and mental health differs when stratified by gender. We gave a primary exposure role to age, different from other variables that we treated as confounders, as it plays both an important biological role on mental health and a relevant social role on income distribution. Our original expressions were indeed not clear enough, so we have changed the sentences to better explain our intended meaning (Line 524-527).

Methodological comments

- Given that we can expect anxiety and depression to be at least moderately correlated, why did the authors not opt to jointly model both mental health outcomes in a single multivariate model?

Reply 1-4: Thanks for this relevant methodological remark. The main reason is that we wanted to preserve the full range of information from direct scores given to each questionnaire. We consider that a multivariate model with two ordered-logistic outcomes would introduce many interpretation problems. These include scale and location indeterminacy, difficulty identifying the model due to correlations and unclear threshold relationships, non-intuitive marginal effects, and potential convergence or sampling issues. Therefore, we believe that two separate models is a cleaner and more parsimonious approach for the present case. The correlation between anxiety and depression is well known, and should imply that patterns for PHQ-9 and GAD-7 are similar on the broader scale, which is consistent with our results.

- The authors state in the significance statement section that the present research provides longitudinal evidence for the relationships of interest. Yet, the longitudinal nature of the data does not seem to be fully exploited since the waves are analysed separately. Why did the authors choose to fit distinct mediation models for each wave, while simultaneously restricting the sample only to participants that took part in both waves?

Reply 1-5: Many thanks. We analysed each wave separately because having a temporal factor of only two timepoints in a single model could create more interpretability problems than solutions. Also, the joint waves may also act as factor within each mental health model (e.g. if we had stratified by wave), but it would complicate interpretation of our main variables. Additionally, we would risk facing convergence issues, which we preferred to avoid. Therefore, since we only concerned about events at the beginning and end of the pandemic, we decided to do separate analyses at this two specific time points. However, we would like to see how the same factors influenced mental health in the two waves. To keep consistency as much as possible, we decided not to include the refreshment samples. The very good convergence and excellent fit (predictive capacity) of our models is a positive indication that using separate models is a reasonable approach; though of course not the only valid one.

- The authors provide posterior predictive checks for the different models, however they chose not to report prior predictive checks, why? Similarly, they do not provide an assessment of the influence of the prior information by running a prior sensitivity analysis or using another approach.

Reply 1-6: Many thanks for bringing this up. We did conduct prior predictive checks and we forgot to include them in the supporting information. This is an error on our part, which we have corrected by providing all prior predictive checks in Supporting information 2 (Annex II) with explanations on why we decided to retain our weakly informative priors in light of the general patterns of our prior distributions. A prior sensitivity analysis is also a good option, but we have decided to focus mostly on prior predictive checks, as we believe these already show that our priors are sufficiently sensible in terms of the –well known— distributions which are generally observed and expected for both income and mental health scores distributions (both right-skewed).

Other comments

- p. 2, l. 36: "Waves-1" should be "Wave-1"

Reply 1-7: Thank you, we corrected it (Line 43).

- p. 5, l. 137-141: summarizing the results using the phrase "males in every income group [...] have a higher estimated probability (averaged across age) of declaring no symptoms of depression than females" is a bit misleading since the estimated probability are almost identical for the first income groups across gender (respectively 64.53% and 64.47%).

Reply 1-8: The expression has been clarified. Please see in Line 158-161: “Results from the hierarchical Bayesian mediation model for Wave-1 showed that, except for the lowest income group (Inc1), males in every other income group had a higher estimated probability (averaged across age) of scoring below the minimal threshold for depression symptoms compared to females.”

- p. 6, table 1: In the legend, it is written: "the effect of income corresponds to the probability change of answering “Not at all” (scoring 0) in the PHQ-9 questionnaire from the highest income group (£1,112+) to the lowest (£0-£300)", but since the value reported is positive, shouldn't it be "from the lowest income group to the highest", as in the legend of figure 1C?

Reply 1-9: Our findings indicated that the probability of scoring 0 in PHQ-9 (i.e., showing minimal or no symptoms of depression) in the highest income group (P_high) is higher than that in the low-income group (P_low) (i.e., the richer the less likely to have depression symptoms). Therefore, the probability change from the highest income group to the lowest income group (P_high - P_low) should be positive (Table 1). That means, the probability of showing no depressive symptom increased from the lowest income group (P_low) to the highest income group (P_high). We have tried to make the expressions about this relationship clearer in the legend for all the tables and legends according to the new statistics from the updated models (e.g., legend for Table 1).

Also it would make the results section clearer I think if the authors referred to both the corresponding table and the corresponding figure pane when discussing a quantity that is reported in both types of data summaries (e.g. p. 5 l. 145-147 : "the probability of reporting no depressive symptoms (PHQ-9 score of 0) increases by 12.4 percentage points between the lowest (£0–£300) and highest (£1,112+) income groups, averaged across the observed age range (18–83 years). In contrast, this probability in females increased only 6.2 percentage points.", and the same information is also represented in figure 1C).
Reply 1-10: The corresponding lines have been clarified (Line 171-175).

- p. 8, l. 191: Similarly to a previous remark, using the sentence "This probability for females also increases, albeit slightly less than males" may be a bit misleading when the estimated values are so similar, with massively overlapping 90% HDI.
Reply 1-11: This has been changed based on the results from the adjusted model (Line 213-216), and we have given attention to the overlapping HDIs when interpreting other results.

- p. 15, l. 280: "with" is missing in "remain constant respect to Wave-1"

Reply 1-12: Thanks for noticing this, it has been corrected (Line 321).

- p. 21, l. 451-453: "Moreover, [...] moderated by age." This sentence seems to mix two distinct results and the articulation between them is not clear.
Reply 1-13: We have edited the sentence to make it clearer: “Moreover, although income does not meaningfully mediate the age-mental health relationship, the strength of the association between income and mental health differs across age.” (Line 555-556)

- p. 21, l. 458-459: I may have misunderstood the sentence but "age-related resilience may enhance the effect of income on mental health" seems inconsistent with figures 3 and 6 that show overall that the income effect is more pronounced among younger individuals.
Reply 1-14: Thanks for spotting it out, “enhance” here should have been “reduce” and it has been corrected (Line 561-562).

- p. 22, l. 463-465: "in" word is probably missing in "that is, older age groups greater susceptibility of the COVID-19"
Reply 1-15: The sentence has been re-written to make it clearer: “This pattern may be due to COVID-19 itself: greater susceptibility of the COVID-19 virus among middle-aged and older populations may have induced heightened levels of distress in older age groups. ” (Line 541-543)

- p. 22, l. 467: I find this subsection on the "Age range" limitation hard to understand. Indeed, the authors seem to suggest at the start of the paragraph that it would be beneficial to investigate similar associations in children and adolescents but they cite prior research with gender-specific income effects for old individuals, while the study sample already covers a large range of adulthood (18-84 years old).
Reply 1-16: We intended to emphasise the fact that our result may not generalise to other age groups in general. To make it clearer, we added some more details and tried to emphasise this point to make this submission more logical and easy to follow (Line 574-580). (To correct the text according to this question, we cited Yang et al., 2025.)

- p. 23-24, l. 519-524: Please provide the numbers of participants excluded for each specific reasons.
Reply 1-17: First, we excluded 89 participants (Line 655) who did not take in part of both waves. Then we excluded 65 participants (Line 657) from the non-white ethnicities due to the lack of representation. This has been added to the Line 654-657.

- p. 24, figure 7: the DAG for the mediation model is not properly displayed in the PDF version of the submission I reviewed
Reply 1-18: Thank you very much for pointing this out. This issue was likely due to a macOS/PC compatibility issue. Figure 7 in our initial submission was the DAG, now we have updated our DAG and have uploaded figures separately (which should be visible at the bottom of the file) and a figure caption list (Figure 9). We have tried our best to upload a PDF file which will not cause similar visual errors.

- p. 24, l. 544: In the equation for the gender-specific intercept α₂, I think there should be no indexing by item q.
Reply 1-19: We tried to make the text about this more clear (Line 712). It is an intercept varying over question and gender as different gender may answer different questions in different ways systematically, so it is with the index by item q.

- p. 25, l. 561: I found the arrow between age and income confusing, since the equation just below captures the direct effects of age and income on mental health outcomes.
Reply 1-20: We intended to use it to represent the relationship among age, income and mental health, but it was indeed not clear with out a DAG. Considering that, we deleted it and the description below the equation is supposed to be enough to explain the complex relationship (Line 705).

- The link provided for the github repository (<https://github.com/SimonErnesto/anxiety_depression_income_pandemic_UK>) in the
submission returns an error 404.
Instead, I found that the link <https://github.com/SimonErnesto/anxiety_depression_pandemic_UK> (no `_income_` in the URL) seems to redirect to the proper code repository.

Reply 1-21: We apologise for the mistake. We have provided the correct link for the repository (OSF) that was required by the editor.

**Reviewer #2**: Overall this is a good paper on an interesting topic which is relevant to PLOS Mental Health. The analysis is methodologically sound, the dataset seems appropriate for the empirical task and the findings are innovative. The only issues I have with the paper as it stands are mainly to do with the description of the C19PRC study and the limitations which the data imposes on the empirical model, which need to be addressed in more detail by the authors. I set out the issues below, along with a few other minor issues.

We really appreciate your time and effort in reviewing our manuscript. In our answers below we have attempted to address your concerns as best as possible. We have expanded on the points you have mentioned to be lacking, and we have further discussed the limitations of our analysis. In addition to that, to add higher methodological rigour, we have re-analysed our data via more robust models, and included additional images for clarity. Your comments have greatly helped us to improve the quality of our manuscript.

1. It would be useful to give a bit more information in Section 4 of the paper about the C19PRC study. At the moment it says that the household income data are from 2019 but the income data for Wave 1 were actually collected in March 2020 (at the start of the Covid-19 pandemic) - survey respondents were asked about what their household income was in 2019. So the income data are collected in 2020 rather than 2019, retrospectively.

Reply 2-1: Many thanks for pointing this out. That is correct. We have added this information to our methods section (lines 657-659).

2. Due to the limitations of the C19PRC data the model for the relationship between income, age and various mental health measures does not include some explanatory variables which have been found to be key determinants of mental health in previous research. The two most obvious omitted variables are employment status and housing tenure (or at least housing costs). The absence of these variables does not invalidate the results but should be mentioned as a limitation of the study.

Reply 2-2: We really appreciate this comment. To address this issue, we included the following variables as confounders adjusted in the income and mental health sub-models: education (no-tertiary, tertiary), employment (unemployed, employed), children (no, yes), religion (non-religious, religious), political affiliation (median split: left-leaning, right-leaning), loneliness (does not meet criteria, meets criteria) and housing (does not own house, owns house) (Line 716-723). Amongst these changes we have included both employment status and housing (and other relevant variables) as adjustments in our models. This observation has been of great help to improve the quality of our manuscript; we hope that our re-analyses and updated results and report satisfy your requirement.

3. As I understand it, C19PRC is a panel study. Only 2 waves were used (Wave 1 and 6) - was there any particular reason for not using data from Waves 2 through 5? Also, did the authors investigate the possibility of exploiting the panel structure of the data to estimate more methodologically robust econometric models (e.g. fixed effects or other panel data models).

Reply 2-3: Many thanks for your comment. Our main focus with this study is observing the longitudinal change from the start of the pandemic to its most advanced point as per C19PRC study. Given that, we did not want to focus on the progression of intermediate waves to avoid both derivative reports that were not our main focus and cluttering the study with information we were not targeting. To make this point clearer in the manuscript, we added Line 645-649. In all honesty, we did not consider exploiting the panel structure of the study for making models more robust. Thank you for this suggestion. Although our main goal was to examine relationships between age, gender, income, and mental health measures (thus keeping mental health as our primary focus), we acknowledge the relevance of your point. We have therefore added it to the discussion as part of future research directions (see section 3.4, Implications and Future Directions, Line 612-624).

4. In Figure 3, the relationship between Age and probability of reporting no mental health symptoms seems very different for females and males in Wave 1, but much more similar in Wave 6. Therefore, the Covid-19 pandemic seems to have somehow "equalised" the age/mental health relationship for women and men, whereas in Wave 1 there was a much clearer age/mental health gradient for men compared with women (at every household income level). It would be good if the authors could attempt to explain these differential gender effects in more detail.

Reply 2-4: Thank you very much, this is an important conclusion from our results and we have focused more deeply on it, to the benefit of our manuscript. Although the specific relationship may show a slight difference from before given our updated statistical model, we discussed more about this point in the discussion (Line 545-571). (To address this issue, in addition to the changes made to the Discussion, we have also added a few lines to the Introduction to provide readers with broader context, citing Platt et al. (2016) and the more recent work of Gao et al. (2023).)

5. In Lines 124-126 of the paper, the authors state "income may mediate this relationship [between mental health and age] due to salary and saving capacity increasing with age (though this may not be reflected in household income). Salary should be reflected in household income measures at least to some extent because earnings income is a key component of household income for working age households (although not the only component).

Reply 2-5 : Many thanks for bringing this up. This was indeed incorrect phrasing on our part. Our intention was to express that in some specific cases (i.e. specific households), salary may not outweigh factors such as savings, inheritances, etc. We re-wrote the sentence to make the paragraph clearer and more logical (Line 139-143).

6. Discussion of results in Table 1: The effect of income corresponds to the probability change of answering "not at all" (scoring zero) in the PHQ-9 questionnaire. Was any robustness analysis conducted using a different threshold for PHQ-9, e.g. looking at the probability of scoring zero or one? It's not clear whether a different cut-off point for "depressed/not depressed" on PHQ-9 would make a difference to the results.

Reply 2-6: We appreciate your comment on this respect. We have added two additional figures that compare the posterior distributions and observed data aggregated over PHQ-9 and GAD-7 standard thresholds as used for mental health assessment in the UK (Figure 1 and Figure 5). These should reflect the most commonly used clinical thresholds for GAD-7 and PHQ-9. Related results have also been reported through Results section, such as in Line 161-163.

Two things can be noticed. 1) The directions of the effects are generally preserved when grouped by thresholds, which means that the model is working well in terms of inferential capacity, as it can show directions of the effects at the single score level which are comparable to the threshold level. 2) The data has a strong category imbalance (usual in anxiety and depression scores), which is reflected in the model as “regression to the mean”. For example, given the low base rate of severe depression (1.2% in our sample), the model's predictions for this category are generally imprecise, but the overall model fit (MAE=0.6) and PPCs support the model’s broader predictive capacity and its use for estimating causal effects. Having said this, both the observed and predicted pattern, when grouped by GAD/PHQ thresholds, show patterns consistent with our main results and with the literature. Females tend to show lower proportions for the mild anxiety or no-depression categories than males, and males tend to show an almost linear relationship from lower to higher incomes in all thresholds (increasing for mild/none, generally decreasing for the other categories). This further supports the core of our analysis. Many thanks for encouraging us to dig further into these patterns as this has improved both the consistency and clarity of our results.

7. The paper doesn't discuss the furlough scheme, SEISS (the self-employed income support scheme) or the £20 per month increase in Universal Credit payments, which were the main mechanisms for distributing financial support to employees, self-employed people and households on low incomes respectively during the Covid-19 pandemic. It's a real shame that only 2019 income was reported in the study (at least, as far as I could tell from the write-up - e.g. line 523-524) as it would be very interesting to examine the relationship between *change* in income and change in mental health status during the Covid-19 pandemic. However, the limitations of the C19PRC data make this impossible.

Reply 2-7: Thank you, we had not considered this point. Although it is very relevant, we had strong methodological reasons for only considering household income in 2019. We chose the average household income in 2019 because it reflects the stable socioeconomic status of families just before the pandemic hit (around Wave-1). We focused more on understanding how that relatively stable income level influenced mental health during a financial shock like COVID-19. Therefore, although the effects of income change due to public policy across the pandemic are very interesting and important our current data are not well suited to explore it. However, as we agree it is an important point to consider, We have added a discussion of how these financial support schemes may offer one possible explanation for our results (Line 445-456) (To address this concern, we added a new citation: HM Revenue and Customs, 2023). Also, we have included a new section to our discussion (Line 449-453 & 597-610) where, amongst other things, we discussed the potential implications of SEISS and how similar measures could be of relevance in light of our present results.

8. The sample size of 688 participants is notably smaller than the total number of adults who completed Wave 1 of the C19PRC study in March 2020. Was any attempt made to examine whether this sample selection may have affected the results - e.g. by comparing descriptive statistics for the 688 participants used in this paper to the full set of Wave 1 participants?

Reply 2-8: Many thanks for pointing this out. At the beginning, we kept only participants (n = 688) that have provided data in every single wave (6 in total). However, since we only concerned about events at the beginning and end of the pandemic, and using all waves would complicate interpretation of our main variables and risk facing convergence issues, we decided to use data from participants who attended both Wave-1 and Wave-6 and who were consistent in the report of their household income from 2019 (Line 658-659). That increased the number of participants to 1009, but we missed to change that in our initial submission. Our apologies for the confusion.

However, following comments from the reviewers, we have excluded the participants of the non-white ethnicities due to their small proportion of (6.4%) of the total sample. In total, our sample is now 939 participants (Line 657-659).

We did not directly compare the 939 participants to the full set of Wave-1 participants (n = 2025). However, Bayesian methods allow for a reduction in sample size while maintaining robust statistical reliability. Also, we would like to reduce the possible inconsistency between the data source for both Wave-1 and Wave-6 to avoid any differences between the waves were introduced by refreshment/different groups of participants.

We thank the reviewer again for bringing up this relevant point, it gives us the chance to explain this better and to clarify that.

9. Line 540: where is Figure 7 (the directed acyclic graph showing the causal effect of age on income, income on mental health, age on mental health and the mediation effect of age on mental health? It was missing from the PDF (or in fact it seems to be covered up by lines 531-538 of the text - I could see the very bottom of the Figure).

Reply 2-9: Thank you very much for pointing this out. This issue was likely due to a macOS/PC compatibility issue. Figure 7 in our initial submission was the DAG, now we have updated our DAG and have uploaded figures separately (which should be visible at the bottom of the file) and a figure caption list (Figure 9). We have tried our best to upload a PDF file which won’t cause visual errors.
