# Supplementary material for: A Bayesian approach for exploring the effects of household income on self-reported mental health measures during the COVID-19 pandemic
Source: PLOS Ment Health. 2026 Aug 25;3(8):e0000691. doi: 10.1371/journal.pmen.0000691 (PMC13505949; doi:10.1371/journal.pmen.0000691)
Supplement: Supplementary file 4 [file pmen.0000691.s005.docx]

Dear Pierre,

Thank you very much for your email. We have gone through the following issues carefully and revised our manuscript "A Bayesian approach for exploring the effects of household income on self-reported mental health measures during the COVID-19 pandemic". Please find the point-by-point response below.

Regards,

Xiao, Simon and Etienne

-------------------------------------------------

1. Limitations section (3.3): Please explicitly acknowledge that participants of non-white ethnicities were excluded due to insufficient representation (n = 65), and that the findings therefore apply to the UK population of white ethnicity, as stated in your response to Reviewer 1. This restriction on generalisability should appear in the manuscript itself, not only in the response letter.

Reply 1: To explicitly acknowledge the limitation created by the exclusion of the participants of non-white ethnicities, we have inserted some more discussion in the limitation section (3.3) (l. 580-589)

2. Data availability: The Data Availability Statement cites the OSF repository ([https://doi.org/10.17605/OSF.IO/UYXTC](https://doi.org/10.17605/OSF.IO/UYXTC)), while Section 5 of the manuscript still cites a GitHub URL — the same link Reviewer 1 reported as returning a 404 error. Please ensure both statements consistently point to the correct, functional repository.

Reply 2: Thank you for pointing this out. Our apologies for missing this error. Now it has been corrected (l. 765).

3. Exclusion criteria: The manuscript (Section 4.1) attributes the exclusion of n = 89 participants to unreliable age reports, whereas your response to Reviewer 1 describes them as participants who did not take part in both waves. Please reconcile these descriptions.

Reply 3: I apologise for my mistake made in the response to Reviewer 1. 89 participants were excluded due to unreliable reports of age as written in the manuscript. To make the exclusion criteria clearer, I have added a little more information about the calculation of the participant numbers (l. 661, 664-666).

4. Figures-compliance with journal specifications and legibility: Please ensure that all figures fully comply with the PLOS figure preparation guidelines ([https://journals.plos.org/mentalhealth/s/figures](https://journals.plos.org/mentalhealth/s/figures)), particularly with respect to dimensions and resolution (TIFF files, 300–600 dpi, width between 789 and 2250 pixels at 300 dpi, fonts in Arial, Times or Symbol at 8–12 pt). Beyond formal compliance, several figures suffer from poor legibility in their current form: in the multi-panel figures (notably Figs 1 and 5), the axis labels, tick labels, and in-panel legends are too small to be read comfortably at publication size, and the hatched "observed" bars are difficult to distinguish from the posterior bars, especially where legend text overlaps the plotting area. Please enlarge fonts, increase line weights where appropriate, and reposition or simplify legends so that all elements remain clearly readable when the figures are reproduced at final column or page width. For figures containing many panels or small text, a resolution of 600 dpi is recommended. You may find PLOS's free figure-checking tool helpful in verifying compliance ([https://ngplosjournals.pagemajik.ai/artanalysis](https://ngplosjournals.pagemajik.ai/artanalysis)).

Reply 4: Thanks for the links. All figures have been re-generated to meet the requirements (TIFF files, 300-600 dpi, width of 2250 pixels with fonts in Arial at 8-12 pt). We also tried our best to make the figures more legible - we enlarged the in-panel legends, reduced the overlapping area of text in the plotting area as much as possible, adjusted the line weights, and we adjusted the transparency of the posterior bars to make them easier to compare with the observed bars, whilst keeping all information within the limited space.

5. Proofreading: Please correct remaining typographical errors, including but not limited to: "probabilities for sales" (should read "males", l. 306); the stray character in "84%]5" (l. 162); "minimal or one" (should read "minimal or none", l. 662); "Patient Heath Questionnaire" (l. 102); "females form the second-lowest income group" (l. 454); and "there several gaps" (l. 608). Please also verify the reported male/female counts, which differ slightly between Wave-1 (476/463) and Wave-6 (475/464) despite the sample being identical across waves.

Reply 5: All typos mentioned above and a couple of more typos have been corrected (l. 316, 162, 102, 463, 620).

An explanation about the changed counts of the two genders have also been added in Line 670-672.
