# Supplementary material for: A Bayesian approach for exploring the effects of household income on self-reported mental health measures during the COVID-19 pandemic
Source: PLOS Ment Health. 2026 Aug 25;3(8):e0000691. doi: 10.1371/journal.pmen.0000691 (PMC13505949; doi:10.1371/journal.pmen.0000691)
Supplement: Peer review history

# Peer review: Decision Letter 0

**DOI:** 10.1371/journal.pmen.0000691.r001

**Authors:** Pierre Jacquet

27 May 2026

PMEN-D-26-00124

A Bayesian approach for exploring the effects of household income on self-reported mental health measures during the COVID-19 pandemic

PLOS Mental Health

Dear Dr. Xiao Fu,

Thank you for submitting your manuscript PMEN-D-26-00124, “A Bayesian approach for exploring the effects of household income on self-reported mental health measures during the COVID-19 pandemic”, to PLOS Mental Health.

Your manuscript has now been evaluated by two expert reviewers. Both were supportive of the work and considered that the manuscript addresses a topic of clear relevance to the journal. On the basis of their reports and my own assessment, I am pleased to invite you to submit a minor revision.

The reviewers’ comments are constructive and identify a number of points that should be addressed to improve the clarity, transparency, and interpretation of the manuscript. In particular, the reviewers ask for clarification of aspects of the C19PRC study and the analytical sample, a more explicit discussion of the limitations imposed by the available data, additional justification for the modelling choices, and some refinements to the interpretation and presentation of the results.

Please note that this summary is intended only to highlight some of the main issues raised during review and should not be treated as a substitute for the reviewers’ reports. In preparing your revision, please address all comments raised by both reviewers in full. Your response letter should provide a detailed point-by-point reply to each comment, indicating how the manuscript has been revised and referring to the relevant sections or line numbers. Where you decide not to implement a suggested change, please provide a clear justification.

Provided that the revised manuscript satisfactorily addresses the reviewers’ full set of comments, I expect that it should be suitable for publication without the need for further revision.

I look forward to receiving your revised manuscript and response letter.

Sincerely,

Pierre Olivier Jacquet, PhD

Academic Editor

PLOS Mental Health

Please submit your revised manuscript by Jun 26 2026 11:59PM. If you will need more time than this to complete your revisions, please reply to this message or contact the journal office at mentalhealth@plos.org. When you're ready to submit your revision, log on to https://www.editorialmanager.com/pmen/ and select the 'Submissions Needing Revision' folder to locate your manuscript file.

Please include the following items when submitting your revised manuscript:

- A letter that responds to each point raised by the editor and reviewer(s). You should upload this letter as a separate file labeled 'Response to Reviewers'.
- A marked-up copy of your manuscript that highlights changes made to the original version. You should upload this as a separate file labeled 'Revised Manuscript with Track Changes'.
- An unmarked version of your revised paper without tracked changes. You should upload this as a separate file labeled 'Manuscript'.

Guidelines for resubmitting your figure files are available below the reviewer comments at the end of this letter.

As the corresponding author, your ORCID iD is verified in the submission system and will appear in the published article. PLOS supports the use of ORCID, and we encourage all coauthors to register for an ORCID iD and use it as well. Please encourage your coauthors to verify their ORCID iD within the submission system before final acceptance, as unverified ORCID iDs will not appear in the published article. *Only*  the individual author can complete the verification step; PLOS staff *cannot*  verify ORCID iDs on behalf of authors.

Journal Requirements:

1. We ask that a manuscript source file is provided at Revision. Please upload your manuscript file as a .doc, .docx, .rtf or .tex.

2. Please provide separate figure files in .tif or .eps format.

For more information about figure files please see our guidelines:  LINK

https://journals.plos.org/mentalhealth/s/figures

https://journals.plos.org/mentalhealth/s/figures#loc-file-requirements

3. We have noticed that you have uploaded Supporting Information files, but you have not included a list of legends. Please add a full list of legends for your Supporting Information files after the references list.

4. Thank you for uploading your study's underlying data set. Unfortunately, the repository you have noted in your Data Availability statement does not qualify as an acceptable data repository according to PLOS's standards.

At this time, please upload the minimal data set necessary to replicate your study's findings to a stable, public repository (such as figshare or Dryad) and provide us with the relevant URLs, DOIs, or accession numbers that may be used to access these data. For a list of recommended repositories and additional information on PLOS standards for data deposition, please see https://journals.plos.org/plosone/s/recommended-repositories.

If the reviewer comments include a recommendation to cite specific previously published works, please review and evaluate these publications to determine whether they are relevant and should be cited. There is no requirement to cite these works unless the editor has indicated otherwise.

Please review your reference list to ensure that it is complete and correct. If you have cited papers that have been retracted, please include the rationale for doing so in the manuscript text, or remove these references and replace them with relevant current references. Any changes to the reference list should be mentioned in the rebuttal letter that accompanies your revised manuscript. If you need to cite a retracted article, indicate the article’s retracted status in the References list and also include a citation and full reference for the retraction notice.

[Note: HTML markup is below. Please do not edit.]

Reviewers' comments:

Reviewer's Responses to Questions

**Comments to the Author**

1. Does this manuscript meet PLOS Mental Health’s publication criteria?

Reviewer #1: Yes

Reviewer #2: Yes

**********

2. Has the statistical analysis been performed appropriately and rigorously?

Reviewer #1: Yes

Reviewer #2: Yes

**********

3. Have the authors made all data underlying the findings in their manuscript fully available (please refer to the Data Availability Statement at the start of the manuscript PDF file)?

The PLOS Data policy

Reviewer #1: Yes

Reviewer #2: Yes

**********

4. Is the manuscript presented in an intelligible fashion and written in standard English?

Reviewer #1: Yes

Reviewer #2: Yes

**********

Reviewer #1: This manuscript uses publicly available longitudinal data gathered from a UK sample during the COVID-19 pandemic to examine the relationship between weekly income levels and mental health in the early stage of the health crisis and 20 months later. Widely used standardized self-reported measures for depression and anxiety are used as mental health outcomes. Since income and mental health are known to vary across age and gender, the authors rely on a bayesian hierarchical framework to account for the confounding associations with these two demographic factors. More specifically, they estimate mediation models with direct effects of age on

mental health and indirect effects through income levels, as well as gender-specific parameters for mental health baseline levels and the effects of income and age.

Although the analyses reported suggest a negligible mediation of age by income in all models, consistent negative effects of age and income on depression and anxiety were found. Furthermore, the analytical framework used by the authors allow them to reveal interesting age- and gender-specific patterns for income effects, that seem to have shaped participants' mental health outcomes

throughout the pandemic. In particular, the depression and anxiety reducing effect of higher income was consistently greater in males than females, but showed a marked increase between the beginning of the pandemic and 20 months later which was especially strong for females.

Overall, given the manuscript's robust statistical methodology supported by publicly shared analysis codes, and provided that the authors satisfactorily address the points raised below, I would support its publication in PLOS Mental Health.

---

General comments

- The effects of household income levels on mental healths are said to be explored via a "Bayesian causal model", which is represented by the simple Directed Acyclic Graph (DAG) for the mediation model in figure 7. Yet, in the first sentence of the abstract the authors state that the relationship between income and mental health can be confounded by "age, gender, and other demographic factors". Therefore, do the authors consider that the coefficients they report should be interpreted causally or are there obvious unmeasured confounding variables that likely generate some bias?

- The authors conclude by highlighting the value of the reported evidence to "inform targeted mental health policies and economic support programs for future emergencies". Could they explicit a bit more what type of health policies and/or resource allocation adjustments the age- and gender-specific patterns obtained may call for?

- p. 24, l. 436-438: The authors say "Accordingly, the effect of age on income was not a primary focus in the present research; instead, we examined the joint influence of age and income on mental health, as well as how the effect of income on mental health varies across age.", but this seems in contradiction with the presentation of the analytical strategy in other parts of the manuscript (e.g. lines 110-111 "our goal is to propose a methodological framework for understanding the effects of income on mental health as a potential mediator of age") and the fact that they highlight the novelty of fitting a bayesian causal mediation model (e.g. "Income effects on mental health have been widely demonstrated in the previous literature (see Thomson et al., 2022), but they have not been systematically explored, using bespoke causal models").

Methodological comments

- Given that we can expect anxiety and depression to be at least moderately correlated, why did the authors not opt to jointly model both mental health outcomes in a single multivariate model?

- The authors state in the significance statement section that the present research provides longitudinal evidence for the relationships of interest. Yet, the longitudinal nature of the data does not seem to be fully exploited since the waves are analysed separately. Why did the authors choose to fit distinct mediation models for each wave, while simultaneously restricting the sample only to participants that took part in both waves?

- The authors provide posterior predictive checks for the different models, however they chose not to report prior predictive checks, why? Similarly, they do not provide an assessment of the influence of the prior information by running a prior sensitivity analysis or using another approach.

Other comments

- p. 2, l. 36: "Waves-1" should be "Wave-1"

- p. 5, l. 137-141: summarizing the results using the phrase "males in every income group [...] have a higher estimated probability (averaged across age) of declaring no symptoms of depression than females" is a bit misleading since the estimated probability are almost identical for the first income groups across gender (respectively 64.53% and 64.47%).

- p. 6, table 1: In the legend, it is written: "the effect of income corresponds to the probability change of answering “Not at all” (scoring 0) in the PHQ-9 questionnaire from the highest income group (£1,112+) to the lowest (£0-£300)", but since the value reported is positive, shouldn't it be "from the lowest income group to the highest", as in the legend of figure 1C?

Also it would make the results section clearer I think if the authors referred to both the corresponding table and the corresponding figure pane when discussing a quantity that is reported in both types of data summaries (e.g. p. 5 l. 145-147 : "the probability of reporting no depressive symptoms (PHQ-9 score of 0) increases by 12.4 percentage points between the lowest (£0–£300) and highest (£1,112+) income groups, averaged across the observed age range (18–83 years). In contrast, this probability in females increased only 6.2 percentage points.", and the same information is also represented in figure 1C).

- p. 8, l. 191: Similarly to a previous remark, using the sentence "This probability for females also increases, albeit slightly less than males" may be a bit misleading when the estimated values are so similar, with massively overlapping 90% HDI.

- p. 15, l. 280: "with" is missing in "remain constant respect to Wave-1"

- p. 21, l. 451-453: "Moreover, [...] moderated by age." This sentence seems to mix two distinct results and the articulation between them is not clear.

- p. 21, l. 458-459: I may have misunderstood the sentence but "age-related resilience may enhance the effect of income on mental health" seems inconsistent with figures 3 and 6 that show overall that the income effect is more pronounced among younger individuals.

- p. 22, l. 463-465: "in" word is probably missing in "that is, older age groups greater susceptibility of the COVID-19"

- p. 22, l. 467: I find this subsection on the "Age range" limitation hard to understand. Indeed, the authors seem to suggest at the start of the paragraph that it would be beneficial to investigate similar associations in children and adolescents but they cite prior research with gender-specific income effects for old individuals, while the study sample already covers a large range of adulthood (18-84 years old).

- p. 23-24, l. 519-524: Please provide the numbers of participants excluded for each specific reasons.

- p. 24, figure 7: the DAG for the mediation model is not properly displayed in the PDF version of the submission I reviewed

- p. 24, l. 544: In the equation for the gender-specific intercept α₂, I think there should be no indexing by item q.

- p. 25, l. 561: I found the arrow between age and income confusing, since the equation just below captures the direct effects of age and income on mental health outcomes.

- The link provided for the github repository (https://github.com/SimonErnesto/anxiety_depression_income_pandemic_UK) in the

submission returns an error 404.

Instead, I found that the link https://github.com/SimonErnesto/anxiety_depression_pandemic_UK (no `_income_` in the URL) seems to redirect to the proper code repository.

Reviewer #2: Overall this is a good paper on an interesting topic which is relevant to PLOS Mental Health. The analysis is methodologically sound, the dataset seems appropriate for the empirical task and the findings are innovative. The only issues I have with the paper as it stands are mainly to do with the description of the C19PRC study and the limitations which the data imposes on the empirical model, which need to be addressed in more detail by the authors. I set out the issues below, along with a few other minor issues.

1. It would be useful to give a bit more information in Section 4 of the paper about the C19PRC study. At the moment it says that the household income data are from 2019 but the income data for Wave 1 were actually collected in March 2020 (at the start of the Covid-19 pandemic) - survey respondents were asked about what their household income was in 2019. So the income data are collected in 2020 rather than 2019, retrospectively.

2. Due to the limitations of the C19PRC data the model for the relationship between income, age and various mental health measures does not include some explanatory variables which have been found to be key determinants of mental health in previous research. The two most obvious omitted variables are employment status and housing tenure (or at least housing costs). The absence of these variables does not invalidate the results but should be mentioned as a limitation of the study.

3. As I understand it, C19PRC is a panel study. Only 2 waves were used (Wave 1 and 6) - was there any particular reason for not using data from Waves 2 through 5? Also, did the authors investigate the possibility of exploiting the panel structure of the data to estimate more methodologically robust econometric models (e.g. fixed effects or other panel data models).

4. In Figure 3, the relationship between Age and probability of reporting no mental health symptoms seems very different for females and males in Wave 1, but much more similar in Wave 6. Therefore, the Covid-19 pandemic seems to have somehow "equalised" the age/mental health relationship for women and men, whereas in Wave 1 there was a much clearer age/mental health gradient for men compared with women (at every household income level). It would be good if the authors could attempt to explain these differential gender effects in more detail.

5. In Lines 124-126 of the paper, the authors state "income may mediate this relationship [between mental health and age] due to salary and saving capacity increasing with age (though this may not be reflected in household income). Salary should be reflected in household income measures at least to some extent because earnings income is a key component of household income for working age households (although not the only component).

6. Discussion of results in Table 1: The effect of income corresponds to the probability change of answering "not at all" (scoring zero) in the PHQ-9 questionnaire. Was any robustness analysis conducted using a different threshold for PHQ-9, e.g. looking at the probability of scoring zero or one? It's not clear whether a different cut-off point for "depressed/not depressed" on PHQ-9 would make a difference to the results.

7. The paper doesn't discuss the furlough scheme, SEISS (the self-employed income support scheme) or the £20 per month increase in Universal Credit payments, which were the main mechanisms for distributing financial support to employees, self-employed people and households on low incomes respectively during the Covid-19 pandemic. It's a real shame that only 2019 income was reported in the study (at least, as far as I could tell from the write-up - e.g. line 523-524) as it would be very interesting to examine the relationship between *change* in income and change in mental health status during the Covid-19 pandemic. However, the limitations of the C19PRC data make this impossible.

8. The sample size of 688 participants is notably smaller than the total number of adults who completed Wave 1 of the C19PRC study in March 2020. Was any attempt made to examine whether this sample selection may have affected the results - e.g. by comparing descriptive statistics for the 688 participants used in this paper to the full set of Wave 1 participants?

9. Line 540: where is Figure 7 (the directed acyclic graph showing the causal effect of age on income, income on mental health, age on mental health and the mediation effect of age on mental health? It was missing from the PDF (or in fact it seems to be covered up by lines 531-538 of the text - I could see the very bottom of the Figure).

Subject to the authors being able to address these issues (including additional explanation and corrections where necessary) I would be happy to recommend publication of this paper.

**********

what does this mean?). If published, this will include your full peer review and any attached files.

**Do you want your identity to be public for this peer review?**  If you choose “no”, your identity will remain anonymous but your review may still be made public.

For information about this choice, including consent withdrawal, please see our Privacy Policy

Reviewer #1: No

Reviewer #2: No

**********

[NOTE: If reviewer comments were submitted as an attachment file, they will be attached to this email and accessible via the submission site. Please log into your account, locate the manuscript record, and check for the action link "View Attachments". If this link does not appear, there are no attachment files.]

# Author comment: Author response to Decision Letter 1

**DOI:** 10.1371/journal.pmen.0000691.r002

26 Jun 2026

Attachment Submitted filename: marked_response_to_reviewers.docx

# Editor report: Decision Letter 1

**DOI:** 10.1371/journal.pmen.0000691.r003

**Authors:** Pierre Jacquet

10 Jul 2026

PMEN-D-26-00124R1

A Bayesian approach for exploring the effects of household income on self-reported mental health measures during the COVID-19 pandemic

PLOS Mental Health

Dear Xiao, dear Simon, dear Etienne,

Thank you for submitting your revised manuscript, "A Bayesian approach for exploring the effects of household income on self-reported mental health measures during the COVID-19 pandemic" (PMEN-D-26-00124R1), to PLOS Mental Health, and for your detailed responses to the reviewers' comments.

As you have noted, both reviewers assessed the original submission favourably and recommended publication subject to minor revisions.

I have carefully examined your point-by-point responses and the revised manuscript.

I am satisfied that you have addressed the reviewers' concerns thoroughly and constructively.

In particular, the addition of a confounder adjustment set (education, employment, children, religion, political affiliation, loneliness, and housing) substantially strengthens the causal interpretation of your models and responds directly to the central concerns of both reviewers. The inclusion of prior predictive checks, the new analyses aggregated by clinical PHQ-9 and GAD-7 thresholds, the expanded description of the C19PRC data and its constraints, and the new discussion of pandemic-era financial support schemes (CJRS, SEISS) and policy implications all represent meaningful improvements to the manuscript.

I am therefore pleased to inform you that **your manuscript is accepted in principle**  for publication in PLOS Mental Health.

However, before the manuscript can proceed to production, a small number of editorial adjustments remain necessary.

**So that you are able to upload your corrected files through Editorial Manager, I am formally issuing a decision of Minor Revision, however, this decision reflects workflow considerations only. The manuscript will not be sent back to the reviewers, and the points listed below will be verified by myself upon resubmission.**

1. Limitations section (3.3): Please explicitly acknowledge that participants of non-white ethnicities were excluded due to insufficient representation (n = 65), and that the findings therefore apply to the UK population of white ethnicity, as stated in your response to Reviewer 1. This restriction on generalisability should appear in the manuscript itself, not only in the response letter.

2. Data availability: The Data Availability Statement cites the OSF repository (https://doi.org/10.17605/OSF.IO/UYXTC), while Section 5 of the manuscript still cites a GitHub URL — the same link Reviewer 1 reported as returning a 404 error. Please ensure both statements consistently point to the correct, functional repository.

3. Exclusion criteria: The manuscript (Section 4.1) attributes the exclusion of n = 89 participants to unreliable age reports, whereas your response to Reviewer 1 describes them as participants who did not take part in both waves. Please reconcile these descriptions.

4. Figures-compliance with journal specifications and legibility: Please ensure that all figures fully comply with the PLOS figure preparation guidelines (https://journals.plos.org/mentalhealth/s/figures), particularly with respect to dimensions and resolution (TIFF files, 300–600 dpi, width between 789 and 2250 pixels at 300 dpi, fonts in Arial, Times or Symbol at 8–12 pt). Beyond formal compliance, several figures suffer from poor legibility in their current form: in the multi-panel figures (notably Figs 1 and 5), the axis labels, tick labels, and in-panel legends are too small to be read comfortably at publication size, and the hatched "observed" bars are difficult to distinguish from the posterior bars, especially where legend text overlaps the plotting area. Please enlarge fonts, increase line weights where appropriate, and reposition or simplify legends so that all elements remain clearly readable when the figures are reproduced at final column or page width. For figures containing many panels or small text, a resolution of 600 dpi is recommended. You may find PLOS's free figure-checking tool helpful in verifying compliance (https://ngplosjournals.pagemajik.ai/artanalysis).

5. Proofreading: Please correct remaining typographical errors, including but not limited to: "probabilities for sales" (should read "males", l. 306); the stray character in "84%]5" (l. 162); "minimal or one" (should read "minimal or none", l. 662); "Patient Heath Questionnaire" (l. 102); "females form the second-lowest income group" (l. 454); and "there several gaps" (l. 608). Please also verify the reported male/female counts, which differ slightly between Wave-1 (476/463) and Wave-6 (475/464) despite the sample being identical across waves.

Once these corrections have been made, please submit your revised files through Editorial Manager in the usual way. Provided the points above are addressed, I anticipate proceeding directly to acceptance without further review.

Thank you for choosing PLOS Mental Health for your work. I look forward to receiving the final version of your manuscript.

Kind regards,

Pierre

Academic Editor

PLOS Mental Health

__________________________________

Please submit your revised manuscript by Aug 09 2026 11:59PM. If you will need more time than this to complete your revisions, please reply to this message or contact the journal office at mentalhealth@plos.org. When you're ready to submit your revision, log on to https://www.editorialmanager.com/pmen/ and select the 'Submissions Needing Revision' folder to locate your manuscript file.

Please include the following items when submitting your revised manuscript:

- A letter that responds to each point raised by the editor and reviewer(s). You should upload this letter as a separate file labeled 'Response to Reviewers'.
- A marked-up copy of your manuscript that highlights changes made to the original version. You should upload this as a separate file labeled 'Revised Manuscript with Track Changes'.
- An unmarked version of your revised paper without tracked changes. You should upload this as a separate file labeled 'Manuscript'.

Guidelines for resubmitting your figure files are available below the reviewer comments at the end of this letter.

As the corresponding author, your ORCID iD is verified in the submission system and will appear in the published article. PLOS supports the use of ORCID, and we encourage all coauthors to register for an ORCID iD and use it as well. Please encourage your coauthors to verify their ORCID iD within the submission system before final acceptance, as unverified ORCID iDs will not appear in the published article. *Only*  the individual author can complete the verification step; PLOS staff *cannot*  verify ORCID iDs on behalf of authors.

# Author comment: Author response to Decision Letter 2

**DOI:** 10.1371/journal.pmen.0000691.r004

21 Jul 2026

Attachment Submitted filename: Response_to_editor.docx

# Editor report: Decision Letter 2

**DOI:** 10.1371/journal.pmen.0000691.r005

**Authors:** Pierre Jacquet

29 Jul 2026

A Bayesian approach for exploring the effects of household income on self-reported mental health measures during the COVID-19 pandemic

PMEN-D-26-00124R2

Dear Simon, dear Xiao, dear Etienne,

Thank you for the latest revisions to your article. I am delighted to be able to finally announce its official acceptance.

Congratulations on this superb work!

Best regards.

Pierre O. Jacquet, PhD

Academic Editor

PLOS Mental Health

______________________________________

We are pleased to inform you that your manuscript 'A Bayesian approach for exploring the effects of household income on self-reported mental health measures during the COVID-19 pandemic' has been provisionally accepted for publication in PLOS Mental Health.

Before your manuscript can be formally accepted you will need to complete some formatting changes, which you will receive in a follow up email. A member of our team will be in touch with a set of requests.

Please note that your manuscript will not be scheduled for publication until you have made the required changes, so a swift response is appreciated.

IMPORTANT: The editorial review process is now complete. PLOS will only permit corrections to spelling, formatting or significant scientific errors from this point onwards. Requests for major changes, or any which affect the scientific understanding of your work, will cause delays to the publication date of your manuscript.

If your institution or institutions have a press office, please notify them about your upcoming paper to help maximize its impact. If they'll be preparing press materials, please inform our press team as soon as possible -- no later than 48 hours after receiving the formal acceptance. Your manuscript will remain under strict press embargo until 2 pm Eastern Time on the date of publication. For more information, please contact mentalhealth@plos.org.

Thank you again for supporting Open Access publishing; we are looking forward to publishing your work in PLOS Mental Health.

Best regards,

PLOS Mental Health
